# Efficacy, moderators and mediators of cognitive behavioural analysis system of psychotherapy (CBASP) versus behavioural activation (BA) in persistently depressed treatment-resistant inpatients: study protocol for the multicentre, randomised controlled changePDD trial

**DOI:** 10.1136/bmjopen-2025-107051

**Published:** 2026-04-01

**Authors:** Eva-Lotta Brakemeier, Jan Philipp Klein, Johannes Zimmermann, Maike Hollandt, Matthias A Reinhard, Sabrina Boger, Luisa Daldrup, Luk Eldem, Philippa Gebhardt, Susanne Heinrich, Matthias Hirsmueller, Jacob Millerowski, Martina Richter, Isabelle C Ridderbusch, Svenja Suerig, Lennart Schroeter, Kerstin Velten-Schurian, Stefan Engeli, Amelie Witte, Malek Bajbouj, Andreas J Fallgatter, Kai G Kahl, Tilo Kircher, Stephan Köhler, Alexandra Philipsen, Martin Walter, Johannes Wolf, Anne Guhn, Ulrich Schweiger, Jürgen Hoyer, Dennis Gutzmer, Stephan Mauersberger, Selin Demir, Sarah Stapel, Jonas Hallgans, Cornelius Schüle, Christian Frischholz, Ivo Heitland, Stephanie Rek, Andrea Jobst, Ina Kluge, Silke Lux, Nils Opel, Svenja Orlowski, Christina Reinert, Henrike Voelz, Isabel M Berwian, Helge Frieling, Hannah B Maier, Henrik Walter, Eva Fassbinder, Tim Kaiser, Jonathan Kanter, John Swan, Pim Cuijpers, Martin Hautzinger, Philipp Sterzer, Frank Padberg

**Affiliations:** 1 Department of Clinical Psychology and Psychotherapy, University of Greifswald, Greifswald, Germany; 2 Department of Psychiatry, Psychosomatics and Psychotherapy, Lübeck University, Lübeck, Germany; 3 Department of Psychology, University of Kassel, Kassel, Germany; 4 Department of Psychiatry and Psychotherapy, University Hospital LMU Munich, Munich, Germany; 5 Partner Site Munich-Augsburg, DZPG (German Center for Mental Health), Munich, Germany; 6 Department of Psychiatry and Neurosciences, Charité Universitätsmedizin Berlin, Berlin, Germany; 7 Department of Psychiatry and Psychotherapy, University Hospital Bonn, Bonn, Germany; 8 Department of Psychiatry, Social Psychiatry and Psychotherapy, Hannover Medical School, Hannover, Germany; 9 University Hospital for Psychiatry and Psychotherapy, University of Tuebingen, Tuebingen, Germany; 10 Department of Psychiatry and Psychotherapy, University Hospital Jena, Jena, Germany; 11 Department of Psychiatry and Psychotherapy, Philipps-University Marburg, Marburg, Germany; 12 Coordinating Center for Clinical Studies (KKS), University Medicine Greifswald, Greifswald, Germany; 13 Partner Site Berlin, DZPG (German Center for Mental Health), Berlin, Germany; 14 Partner Site Tuebingen, DZPG (German Center for Mental Health), Tuebingen, Germany; 15 Translational Psychiatry Lab, University Psychiatric Clinics Basel, Basel, Switzerland; 16 Department of Clinical Psychology and Psychotherapy, University of Dresden, Dresden, Germany; 17 Research Department of Clinical, Educational and Health Psychology, University College London, London, UK; 18 Department of Psychiatry, Social Psychiatry and Psychotherapy, Medizinische Hochschule Hannover, Hannover, Germany; 19 Social Psychiatry and Psychotherapy, Hannover Medical School, Hannover, Germany; 20 Department of Methods and Evaluation/Quality Assurance, Free University Berlin, Berlin, Germany; 21 Department of Psychology, University of Washington, Seattle, Washington, USA; 22 Department of Psychiatry, State University of New York, University at Buffalo, Buffalo, New York, USA; 23 Department of Clinical, Neuro and Developmental Psychology, Vrije Universiteit Amsterdam, Amsterdam, The Netherlands; 24 Department of Psychology, Clinical Psychology, and Psychotherapy, University of Tuebingen, Tübingen, Germany

**Keywords:** Depression & mood disorders, Adult psychiatry, Randomized Controlled Trial

## Abstract

**Introduction:**

Up to 30% of individuals with depression develop persistent depressive disorder (PDD), an often disabling and difficult to treat condition. The Cognitive Behavioural Analysis System of Psychotherapy (CBASP) is the only psychotherapy developed specifically for treating individuals with PDD. While several randomised controlled trials (RCTs) have demonstrated its efficacy in outpatient settings, evidence for its use in inpatient settings remains limited. Pilot studies of CBASP inpatient programmes in Germany have shown promising feasibility and effectiveness; however, no RCTs to date have systematically evaluated their outcomes. This study represents the first RCT to compare the short- and long-term efficacy and safety of CBASP with Behavioural Activation (BA), a first-line psychotherapy for depression, within an intensive multimodal inpatient setting.

**Methods and analysis:**

In this prospective, multicentre, rater-blinded RCT with an active control group, we aim to recruit 396 adults (aged 18–70 years) with treatment-resistant PDD at eight German university hospitals. Participants will be randomly assigned to receive either (1) CBASP or (2) BA within an intensive treatment programme consisting of 10 weeks acute treatment in an inpatient and/or day clinic setting, followed by 6 weeks of outpatient continuation treatment. Primary and secondary outcome assessments will be conducted at multiple time points: baseline (T0), treatment onset (T1), after 5 and 10 weeks of acute treatment (T2, T3), at the end of continuation treatment (T4, week 16) and every 2 months up to week 64 (T5, naturalistic follow-up).

The primary outcome measure will be the change in depression severity, as assessed by the Hamilton Depression Rating Scale (24-item version), after 16 weeks of treatment (from T0 to T4). Secondary outcomes will include response, remission, deterioration and relapse rates, self-reported depression and anxiety symptoms and additional psychological variables. A cost-benefit analysis will evaluate the health-economic benefits of both interventions. Additionally, this RCT will explore personalised treatment selection and mechanisms of change, including potential moderators and mediators of treatment effects. The findings from this trial are expected to provide clinicians with evidence-based guidance on choosing CBASP versus BA for inpatients with treatment-resistant PDD.

**Ethics and dissemination:**

This study has received ethical approval from the ethics committees of all participating university hospitals. All participants will provide written informed consent before enrolment. Study findings will be published in peer-reviewed journals and presented at national and international conferences. We have involved people with lived experience from the earliest pilots onward, using their feedback to refine our study design. Ongoing consultation at conferences and public events has further ensured that our research remains grounded in patient perspectives.

**Trial registration number:**

NCT04996433.

**Strengths and limitations of this study**

- Therapist training and structured supervision ensure consistent fidelity, adherence and competence across sites.
- Standardised ratings and procedures ensure that treatment arms differ only in therapeutic content, not in dose or duration.
- The sample size is adequate for detecting primary outcome effects but may be underpowered for moderator or mediator analyses.
- Ecological momentary assessment is used in addition to traditional outcomes to capture real-time behavioural processes.
- As neither patients nor therapists could be blinded and unspecific effects of the inpatient setting may have influenced outcomes, programme-specific effects cannot be fully disentangled.

## Introduction

### Scientific background and study rationale

 Persistent depressive disorder (PDD), as operationalised in the fifth edition of the Diagnostic and Statistical Manual of Mental Disorders (DSM-5), affects 20%–30% of individuals living with depression, with an estimated lifetime prevalence ranging from 3% to 6%.^1 2^ PDD imposes a significant burden, encompassing psychosocial impairment, mental and somatic comorbidities, suicidality and treatment resistance (TR).^2^,^6^ A substantial number of individuals with PDD require hospitalisation, and approximately half of all inpatients with depression present chronic forms.^47^,^10^

The Cognitive Behavioural Analysis System of Psychotherapy (CBASP) is the only psychotherapy approach developed specifically for the treatment of PDD^11^ and has demonstrated efficacy as outpatient treatment in an increasing number of randomised controlled trials (RCTs)^12^,^16^; with further support from reviews and meta-analyses.^17 18^ However, the optimal frequency, intensity and duration of CBASP for achieving meaningful and long-lasting effectiveness in the challenging-to-treat PDD population have yet to be studied, and there are no RCTs investigating the efficacy of CBASP in institutional settings.

In Germany in 2023, approximately 260 000 individuals living with depression received inpatient treatment, and about 30% would probably meet the criteria of PDD, resulting in a substantial economic burden of about one billion Euro per year.^19^ Moreover, PDD represents a major cost driver in depression care worldwide.^20^ Inpatient settings provide the opportunity to deliver psychotherapy at a higher intensity, combining individual and group therapy with multimodal components delivered by an inter-professional team (for a comprehensive review, see Brakemeier *et al*^21^). A recent meta-analysis demonstrated low to moderate and sustained effects of psychotherapy for patients with depression in institutional settings.^22^ Notably, the number of studies was limited, and a substantial number of trials had a high risk of bias. Additionally, it was challenging to distinguish between chronically and non-chronically depressed patients across the studies.

For inpatients with PDD, there is a lack of evidence-based treatments.^10 23^ Some studies suggest that despite an overall reduction in depressive symptoms with extended psychotherapeutic inpatient treatment,^24^ PDD inpatients achieve lower response rates, express higher treatment dissatisfaction and are more likely to relapse after discharge compared with non-chronic depressed inpatients.^7 24 25^ Given that many inpatients do not respond to standard treatments, there is an urgent need for new treatment-phase programmes that integrate acute and continuation treatments to address TR in PDD.^10 23^

In recent years, CBASP has been adapted into a manualised multimodal inpatient and day-patient programme for severely affected inpatients with PDD and TR.^26^ Naturalistic studies support the feasibility and effectiveness of these programmes in PDD.^27^,^32^

Thus, we have initiated an RCT to investigate the efficacy and safety of an intensive multimodal CBASP programme in comparison to a standard evidence-based psychotherapy for depression. For this study, we selected Behavioural Activation (BA), a third-wave variant of cognitive behavioural therapy (CBT),^33 34^ as the comparator. BA has demonstrated efficacy comparable to standard CBT in severely depressed patients.^35^,^40^ Additionally, BA is relatively easy to train, particularly for non-therapists,^41^ and is more practical to implement in inpatient settings.^42^ Notably, BA is recommended as a first-line treatment for depression in the National Institute for Health and Care Excellence (NICE) guidelines (https://www.nice.org.uk/guidance/ng222).

Moreover, our study will examine individual differences in psychotherapy response and mechanisms of change in order to address the classical question ‘What works for whom and why?’.^43^ The significance of this question is underscored by a meta-analysis of RCTs on psychotherapy for depression, which revealed higher outcome variance in the intervention groups compared with control groups, indicating that individuals may respond differently to various interventions.^44 45^ Additionally, an increasing number of psychotherapy researchers have advocated for a more comprehensive examination of the mechanisms underlying the effectiveness of psychotherapy. For instance, Cuijpers^46^ asserted that neither mediators nor mechanisms of action have been firmly established for any therapy due to methodological challenges. Therefore, this study aims to investigate potential moderators and mediators of the treatment effect. The detailed background and descriptions of the selected moderators—child maltreatment^47 48^ and Brain-derived neurotrophic factor (BDNF) methylation^49^—and mediators—interpersonal problems and activities^33 50 51^—can be found in Study Protocol Version 4.0 (available at https://clinicaltrials.gov). This protocol also outlines our two add-on studies: ecological momentary assessment study using an interpersonal activation diary and an effort task assessing behavioural effort and reward.

Beyond effects of acute treatment, previous RCTs on CBASP showed therapeutic efficacy for up to 1 year.^14 16^ Thus, this study will include follow-up visits up to 48 weeks after the 16-week treatment in the main study phase. Finally, cost-effectiveness analyses will accompany the RCT in order to compare economic measures for both CBASP and BA.

### Study aims and hypotheses

This study is the first RCT aiming to compare the short- and long-term efficacy and safety of CBASP with BA, a first-line psychotherapy for depression, within an intensive multimodal inpatient setting.

#### Primary

We hypothesise that CBASP will be more effective than BA implemented as a 16-week multimodal treatment phase programme (inpatient/day clinic followed by outpatient treatment) for patients with PDD and TR in reducing depressive symptoms measured repeatedly over 16 weeks by blinded clinician raters using the Hamilton Depression Rating Scale (24-item version, HDRS-24).

#### Secondary

We further hypothesise that CBASP will demonstrate superior effects compared with BA in terms of response, remission and deterioration rates, lower drop-out rates, reduced relapse rates and decreased severity of self-reported symptoms of depression and anxiety. Additionally, we expect CBASP to result in greater improvements in quality of life and various psychopathological domains.

#### Further aims

Moderator analyses will examine whether child maltreatment or methylation of exon IV of the BDNF gene has an impact on the differential efficacy of the treatments. Regarding mediator analyses, we will examine whether the differential efficacy of the treatments can be explained by treatment-specific changes in interpersonal problems or activity levels. A follow-up survey 48 weeks after the end of the interventions is intended to provide valuable results regarding the long-term outcome of the treatments. Finally, we will conduct cost-benefit analyses to assess the economic implications of both interventions.

## Methods and analysis

This protocol has been developed in accordance with the Standard Protocol Items: Recommendations for Interventional Trials (SPIRIT) 2013 statement^52 53^ and the SPIRIT-guidelines for reporting outcomes in trial protocols.^54^ Please refer to the online supplemental material 1 for the SPIRIT-Outcomes 2022 Checklist (for the combined completion of SPIRIT 2013 and SPIRIT-Outcomes 2022 items).^54^ The trial has been registered at https://clinicaltrials.gov.

### Trial design

**Figure 1:**
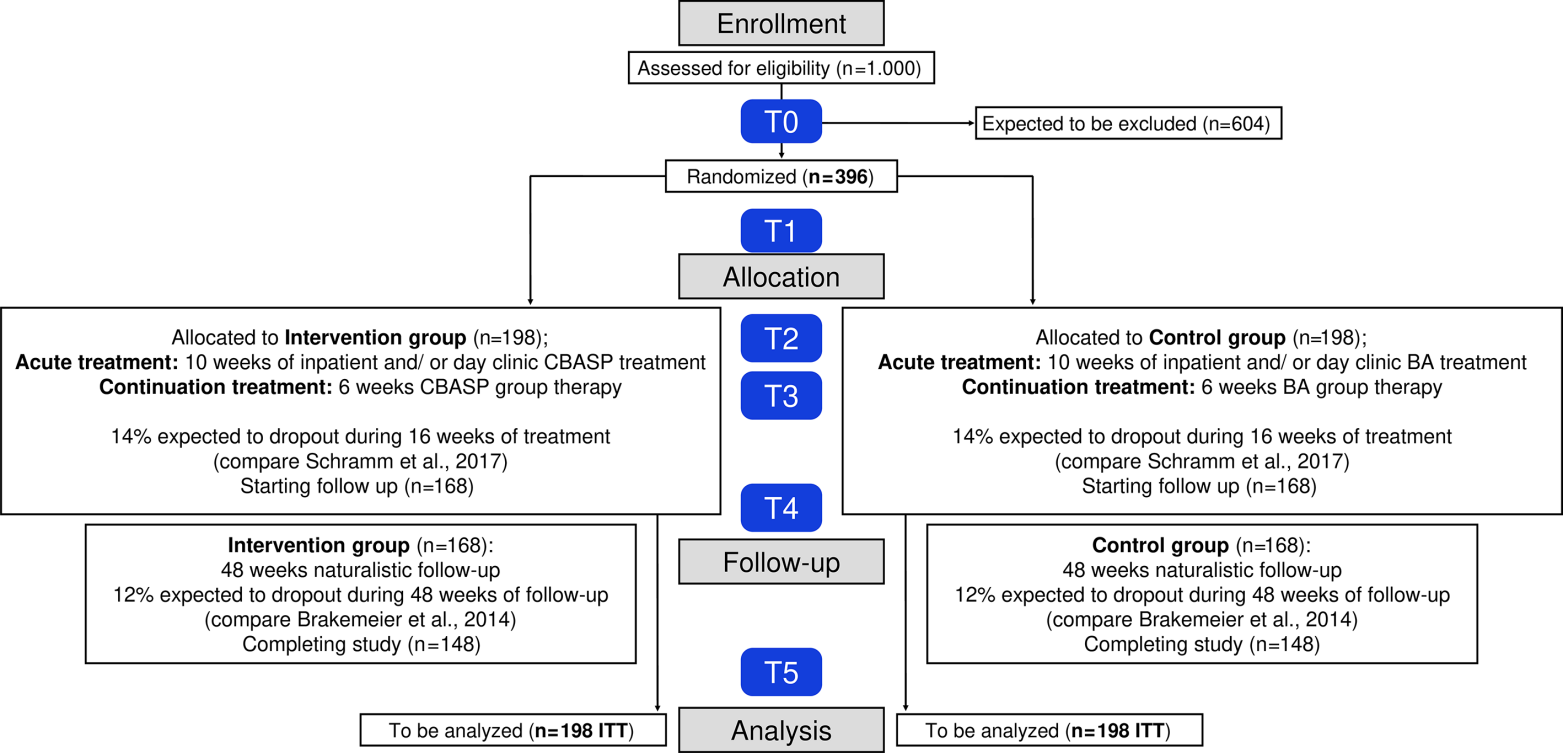
Study flow chart according to CONSORT. BA, Behavioural Activation; CBASP, Cognitive Behavioural Analysis System of Psychotherapy; CONSORT, Consolidated Standards of Reporting Trials; ITT, intention-to-treat.

This is a prospective, multicentre, rater-blinded RCT with a parallel group design and an active control group examining the superiority of CBASP in comparison to BA. Figure 1 presents an overview of the study design.

### Study setting

Study participants are recruited particularly from the waiting lists, depression outpatient clinics and inpatient wards of the eight study centres (university hospitals/departments) located in Germany. Additionally, patient recruitment is facilitated through advertising efforts such as flyers, study websites and advertisements on other relevant websites. We do not aim at a specific sex distribution within the treatment groups, as no gender-specific differences in the efficacy and safety of CBASP or BA are anticipated.

The first study sites were initiated in July 2021, and the study is anticipated to reach its conclusion by February 2028. Initially, we anticipated a 30-month recruitment period, with an estimated recruitment rate of approximately 2.2 patients per month per study site. However, due to the challenges imposed by the COVID-19 pandemic, particularly affecting the study sites, and the persistently lower recruitment rate even after the pandemic subsided, it was necessary to extend the recruitment period to 50 months. Additionally, we expanded the study by incorporating two more study sites, augmenting the original number of 6.

An estimated total of n=1000 patients, aged 18–70, with PDD according to DSM-5 will be screened for eligibility at eight recruiting centres to randomise n=396 participants. The sample size and assumed dropout rate of 14% during treatment have been determined based on the study by Schramm *et al*.^16^ and earlier pilot trials^27^,^29^ of the inpatient treatments in patients with PDD. In particular, we expected that 5% of the patients would drop out of the study directly after randomisation (defined as ‘non-starter’), and a further 10% would drop out of treatment (defined as ‘treatment dropout’) or would drop out of the study during treatment (defined as ‘study dropout’). Therefore, we expect 95%×90%=85.5% of randomised patients to have HDRS-24 non-missing in week 16.

### Eligibility criteria

The main inclusion criteria for the ChangePDD trial are as follows:

1. Age between 18 and 70 years.
2. Primary diagnosis of PDD based on DSM-5 criteria (codes 300.4, 296.2x, 296.3x), confirmed with the Diagnostic Interview for Mental Disorders (DIPS).
3. HDRS-24 score of 20 or higher.
4. TR is defined by the Antidepressant Treatment History Form—Short-Form^55^ or medication intolerance or one psychotherapy (at least 25 sessions in the current episode conducted by a certified therapist).
5. Sufficient proficiency in German.
6. Written informed consent.

The main exclusion criteria are as follows:

1. Diagnosis of bipolar I or II disorder.
2. Active substance use disorders with less than 6 months of abstinence.
3. Diagnosis of schizophrenia spectrum and other psychotic disorders.
4. Presence of antisocial personality disorder.
5. Acute suicidality, indicated by HDRS-24 item 3 score >2 or agreement with Columbia-Suicide Severity Rating Scale item 4 and/or item 5.
6. Previous CBASP or BA treatment within the last year.
7. Inability to tolerate CBASP or BA, such as due to organic brain disorders or severe cognitive deficits.
8. Inability to participate in day clinic or outpatient continuation treatment.

### Interventions

Participants will be randomised once they have completed baseline assessment and allocated to one of the two trial arms (at a 1:1 ratio), that is, the CBASP or the BA treatment arm. Both CBASP and BA are administered as treatment-phase programmes, consisting of a 10-week inpatient and/or day clinic acute treatment, followed by a 6-week outpatient continuation group treatment, all in combination with standardised and guideline-based pharmacotherapy. Both treatments will adhere to a standardised treatment manual (see below).

During the 10-week inpatient and/or day clinic phase, all patients in both programmes will receive the following interventions each week:

- Two individual therapy sessions (each lasting 50 min).
- Two group therapy sessions (each lasting 100 min).
- One nurse contact (therapeutic exchange with a nurse) lasting 25 min per contact.
- One exercise therapy session lasting 75 min.

During the 6-week outpatient continuation phase, patients will receive the following treatment on a weekly basis:

- One group therapy session lasting 100 min.

In total, patients will receive 20 individual therapy sessions, 26 group therapy sessions, 10 nurse contacts and 10 exercise therapy sessions. This treatment ‘dosage’ is similar to or even higher than that used in previous trials of CBASP^16^ and BA.^37^ In cases where treatment sessions are cancelled, for instance, due to illness or vacation, they can be rescheduled at the next available opportunity, including during the 6-week continuation phase. The key goal is to ensure that the target dosage is reached by the end of the 16-week treatment period. Patients who drop out of the study treatment can receive the standard treatment of the corresponding ward, so that clinically there are no disadvantages for the patients due to a dropout.

During the naturalistic follow-up phase (after week 16), patients may receive any further treatments, but CBASP patients should not receive BA therapy and BA patients should not receive CBASP therapy. All treatments received by the patients will be meticulously documented.

Before starting patient recruitment, all study site teams have undergone training in the treatment programmes through supervision and workshops led by BA or CBASP experts. We are collecting demographic and professional information about the therapists using the Therapeutic Attitude Questionnaire, an instrument designed to assess training background, clinical experience, theoretical orientation and conscious therapeutic stance.^56^

Additionally, we have implemented the following treatment fidelity, adherence and competence measures: At each centre, one study patient will be randomly selected for each study therapist. Psychotherapy sessions are videotaped, and three videos will be rated—one from the initial session, one from the middle session and one from the final session. The ratings will be conducted by trained master’s level students, under the supervision of CBASP and BA experts. In addition, 5% of these videos will also be rated by BA and CBASP experts to assess inter-rater reliability. Competence and adherence ratings are based on scales used in previous studies for CBASP and BA, developed in line with the established Cognitive Therapy Scale,^57^ which assesses psychotherapeutic competencies in cognitive therapy. Our adapted instruments are referred to as CAR (Competence and Adherence Rating), specifically BA-CAR and CBASP-CAR. We have established a minimum cut-off to define sufficient therapist adherence, while competence is reflected by the total score achieved (the higher the score, the greater the competence). All videos will be rated using both the CBASP-CAR and BA-CAR rating scales, allowing us to examine whether the two therapeutic approaches differ in their use of techniques and in their therapeutic stance or relationship-building.

#### Conduct of the interventional arm (CBASP)

The CBASP programme will follow a standardised treatment manual.^26^ CBASP was developed specifically for the treatment of chronic forms of early-onset depression. It is based on the assumption that patients with chronic depression experience impaired interpersonal functioning as a result of child maltreatment. The inability to detect the effects of their own behaviour on other people is assumed to increase the likelihood of social isolation or interpersonal conflicts, which in turn worsen depressive symptoms. Thus, CBASP involves specific interpersonal techniques to help patients recognise the consequences of their behaviour and to acquire interpersonal skills to interact more effectively with others. Further specific techniques are applied within the therapeutic relationship to target dysfunctional interpersonal behaviour using therapeutic self-disclosure in a disciplined and personal way (Disciplined Personal Involvement), for example, Contingent Personal Reactivity and the Interpersonal Discrimination Exercise. In this study, CBASP treatment during the inpatient and/or day clinic phase is conducted in three stages. During the introduction (weeks 1 and 2), the impact of the patients’ Significant Other History (SOH) on current interpersonal functioning is analysed and exemplified by one Transference Hypothesis each, for the individual therapist, the multiprofessional team and fellow patients. The information from the introduction phase is shared with the team to identify and anticipate ‘hot spot’ situations, in which patients can achieve interpersonal healing of earlier trauma by significant others. During the main treatment stage (weeks 3–8), interpersonal skills for everyday social situations are acquired through Situational Analyses (SAs) including role play and work with Kiesler’s Circle. During the last stage (weeks 9 and 10), relapse prevention includes the preparation of difficult social situations at home through future SAs and the reflection on correcting interpersonal experiences made with the therapist, team members and fellow patients against the background of the SOH. The group therapy during the outpatient continuation phase will focus mostly on SAs.

#### Conduct of the control arm (BA)

The BA programme will follow a standardised treatment manual.^34^ BA is an empirically supported ‘3rd wave’ version of CBT for depression. It is based on the assumption that a lack of environmental reinforcement and negatively reinforced avoidance behaviours contribute to depression. Thus, BA involves specific techniques to identify and reduce avoidance behaviours as well as increase environmental reinforcement, that is, activity monitoring, assessment of life goals and values, activity scheduling, skills training, relaxation training, contingency management, procedures targeting verbal behaviour and procedures targeting avoidance.^58^ Paralleling the intervention arm, BA is scheduled in three stages during the inpatient and/or day clinic phase. During the introduction (weeks 1 and 2), baseline activities, avoidance behaviour, life goals and values are assessed in order to establish an individual disease model and to set up treatment goals. The model and treatment goals are shared with the multiprofessional team to support and motivate the patients in their efforts to increase their activity level. During the main treatment stage (weeks 3–8), activities are scheduled based on an individual hierarchy and continuously monitored through daily and weekly protocols. To increase the likelihood of successful completion of activities, failures in setting up activities are analysed and rescheduled by incorporating contingency management, context sensitivity, mindfulness training, skills training or other procedures that address avoidance behaviour. The last stage (weeks 9 and 10) is dedicated to relapse prevention, including the preparation for difficult situations. The group therapy during the outpatient continuation phase will focus mostly on supporting self-activation and troubleshooting.

#### Permitted further treatments and services including medication

It is clearly defined which additional relevant concomitant treatments and services are permitted for study patients: standardised physiotherapy group (up to 3/weeks), standardised occupational therapy group (up to 3/weeks), algorithm-based study medication, crisis intervention, relaxation group, mindfulness-based group offered by nurses, patient café, excursion, morning exercise, open patient group and social counselling. The following interventions are not allowed for study patients: mindfulness-based groups offered by therapists and all further psychotherapeutic or non-psychotherapeutic group offers or individual sessions. The ward teams monitor participation closely, and any patient who receives one of these excluded treatments is withdrawn from the study and counted as a drop-out. If a protocol breach is discovered later on, it is logged as a protocol violation. For the permitted treatments/services, particular attention is paid to ensure that study patients do not receive a higher dosage of concomitant interventions in either group. A detailed list including frequency and duration of treatments and services can be found in the Study Protocol V.4.0.

Concerning concomitant medication, most patients will be on antidepressant medication at study entry due to the severity of illness. According to a shared decision process, patients will receive an algorithm-based antidepressant medication following the Guidelines on Unipolar Depression at the time of study initiation.^9^

In case of non-response:

- First line: optimising dosage as needed.
- Second line: augmentation with lithium.
- Third line: augmentation with second generation antipsychotics (ie, Quetiapine, Aripiprazole, Olanzapine or Risperidone) or evidence-based combination with a second antidepressant (combination of selective serotonin reuptake inhibitors, serotonin norepinephrine reuptake inhibitors or tricyclic antidepressants with Mianserine, Mirtazapine or Trazodone).
- Fourth line: change of antidepressant.

Only the following medication is allowed as psychopharmacological rescue medication:

- Zopiclone (on demand up to 7.5 mg/day orally) or Quetiapine (on demand up to a dose of 50 mg/day orally) for sleep disturbances.
- Promethazine (on demand up to 75 mg/day orally) or Quetiapine (on demand up to 50 mg/day orally) for agitation.

This algorithm is in place during the acute phase of the study until the primary endpoint (ie, week 16), thereafter (ie, during the follow-up period) all patients can seek further psychopharmacological treatment according to their clinical needs. Medication is documented in the electronic case report form (eCRFs).

### Participant timeline

Online supplemental table 1 illustrates the frequency and schedule of study visits. The screening phase consists of pre-screening (screening for eligibility via telephone contact) and baseline (T0) comprising in total four sub-visits (V1a–V1d; for a detailed plan see Study Protocol 4.0). The baseline visit is conducted to assess eligibility, obtain informed consent (see onlinesupplemental material 2  3 for the German and English version) and establish baseline measurements. Following informed consent, clinical interviews and self-rating questionnaires are administered. Eligible patients are then randomly assigned to one of the two study arms. Note that all efforts will be made during the informed consent procedure to include only patients who are willing to accept both arms and to continue study visits until W64/T5 irrespective of compliance.

Additional study visits are scheduled as follows: at treatment onset (T1, week 1, V2), at six different time points during the 10-week inpatient and/or day clinic acute phase (week 2, V3; week 4, V4; week 5, V5/T2; week 6, V6; week 8, V7; week 10, V8/T3), at three time points during the continuation phase (week 12, V9; week 14, V10; week 16, V11/T4) and at six time points during the naturalistic follow-up (week 24, V12; week 32, V13; week 40, V14; week 48, V15; week 56, V16; week 64, V17/T5).

### Outcomes

Online supplemental table 2 presents the characteristics and definitions of primary, key secondary and further exploratory endpoints. The primary outcome variable is the change in HDRS-24 total score^59 60^ from baseline to week 16. The HDRS-24 is a frequently used and well-validated clinician-rated measure of depression severity.^61^

The following measures serve as key secondary outcome variables: response (at least 50% reduction of the HDRS-24 score as compared with baseline), remission (HDRS-24 score of 10 or less), deterioration (at least six points increase in HDRS-24 compared with baseline^62^), depressive symptoms as measured by the Inventory of Depressive Symptomatology-Self Report (IDS-SR),^63^ general distress (Brief Symptom Inventory, BSI^64^), global functioning (Global Assessment of Functioning, GAF^65^), quality of life (WHO Quality of Life^66^), relapse at the end of the follow-up period (defined as rehospitalisation for symptomatic worsening and/or a combination of an increase in HDRS-24 from discharge of equal or greater than 10 points and a current HDRS-24 score of equal or greater than 18 points at T5) and dropout from treatment. To examine the health economic benefits of this intervention, we will also examine the societal costs of illness as a secondary outcome through a cost interview which will cover the direct medical costs (eg, inpatient stays, doctor’s visits, emergency treatment, etc), the direct non-medical costs (eg, assisted living, informal care provided by relatives) and the indirect costs (eg, days of incapacity to work, disability, unemployment).^67^ In addition, moderator and mediator variables as well as exploratory outcomes are listed in online supplemental table 2.

All clinical raters receive comprehensive training in all assessment instruments (Diagnostic Interview for Mental Disorders, DIPS; HDRS-24; GAF; Mini-International Classification of Functioning, MINI-ICF; Impact Message Inventory revise, IMI-R; cost interview). In the case of HDRS-24, raters undergo a rigorous training process that begins with a 3-hour web-based training session, followed by an evaluation of three standardised videotaped HDRS-24 interviews. Candidates are approved as study raters only after successfully completing these ratings with a deviation of less than 3 points from the standard. HDRS ratings will be performed by clinical raters blinded to both treatment allocation (CBASP vs BA). Ratings are videotaped for quality reasons.

Of note, as part of our study, we use an eCRF for the entry and storage of all data generated within the ChangePDD project (see section Data Management).

### Safety

Negative effects including side effects of psychotherapy will be measured at weeks 5, 10, 16 and 64 with the Side Effects of Psychological Interventions Process Scale. (Serious) Adverse events (AEs and SAEs) will be continuously monitored via a standardised questionnaire; an AE is defined as any disadvantageous incident that occurs in a person receiving psychotherapeutic intervention, regardless of possible associations with the treatment received.

The following AEs are defined for the study ChangePDD:

- Exacerbation of symptoms, for example, generalisation of symptoms (clinical judgement).
- Appearance of new symptoms.
- Appearance of passive suicidal ideation.
- Appearance of active suicidal plans or intentions.
- Occurrence of problems in the patient-therapist relation.
- Further disadvantageous incidents as assessed by the therapist.

Adverse treatment reaction (ATR) is defined as any AE that is due to the intervention. The determination of causality of AEs is made by the therapists and is supervised and controlled by the principal investigator (PI) and Co-PIs and the Data and Safety Monitoring Board (DSMB).

SAEs and Serious Adverse Treatment Reactions (SATRs) are defined as an AE or ATR resulting in:

- Death.
- Life-threatening event, for example, a suicide attempt.
- An incident requiring hospitalisation.
- An incident leading to significant or permanent disability or invalidity.

(S)AEs and (S)ATRs are regularly assessed and documented using an AE eCRF. In the eCRF, the corresponding therapist or delegate is asked to describe any AE, its duration (start/end date), intensity (mild, moderate, severe), assessment of causality (treatment related, probably related, unlikely related, not related, not assessable), the actions taken and the outcome of the action taken. In addition, the corresponding therapist is asked to assess whether the documented AEs and ATRs are judged serious (SAE, SATR). In the case of changing the individual therapists, the reasons for this change are documented in a separate form. AE documentation is monitored during the study as part of the regular monitoring conducted by the Coordinating Center for Clinical Studies (KKS) Greifswald. SAEs and SATRs must be reported electronically (via fax or e-mail) to the PI and the KKS Greifswald immediately within 24 hours using a SAE report form that provides further details on the incident.

Patients who withdraw from study interventions due to one or more of the above-mentioned AES or SAEs will be followed up in accordance with good clinical practice until a solution is found or the event is no longer considered clinically significant. Patients who drop out for other reasons receive the standard treatment of the corresponding ward, so that clinically there are no disadvantages for the patients due to a dropout.

### Randomisation and blinding

Patients are randomised into either the CBASP group or the BA group. Block randomisation with stratification according to severity of depression and participating site is used. The participating sites are Berlin, Hannover, Lübeck, Marburg, Munich and Tübingen, as well as the later included sites Bonn and Jena. The allocation ratio is set at 1:1 and is performed by the KKS in Greifswald. Patients are randomised using pre-generated lists. To ensure balance between the two treatment groups within each participating site and in relation to depression severity, stratified permuted blocks are used to generate the randomisation lists. For the latter, we implemented an HDRS-24 score of at least 27 points as the binary cut-off criterion, mirroring the mean baseline value of the HDRS-24 in the study conducted by Schramm *et al*.^16^ Furthermore, we introduce random block sizes of 4, 6 or 8 to heighten the unpredictability of treatment assignment. The selection of permutations within a block is entirely randomised, and the number of treatment groups is evenly distributed within each block. To ensure allocation concealment, the chief coordinator of each site notifies the KKS if a patient has been successfully screened at the site, along with the patient’s HDRS-24 score. The site chief coordinator is then notified by the KKS of the randomisation’s outcome.

Due to the nature of the interventions, achieving blinding of patients and therapists with respect to the treatment is unfeasible. However, efforts are made to ensure that patients and treatment teams on the wards remain unaware of the primary study hypothesis. They are informed that it is currently unknown which of the two psychotherapy programmes is more effective for treating patients with PDD and TR. Ideally, the same blinded clinical rater (student assistants at each participating site) will conduct patient assessments at all timepoints. Procedures to maintain blinding include physically separating the clinical raters from the study wards, instructing patients not to disclose information that could reveal their allocation, and having backup raters available in case unintentional unblinding occurs.

### Sample size calculation

We intended the sample size to be large enough so that the power to detect a difference in the changes between treatment groups (CBASP vs BA) of at least three HDRS points after 16 weeks is 90% (assuming alpha=0.05). This target effect size is in line with the NICE guidelines,^68^ stating that a difference between treatment groups of at least three points on the HDRS-24 is clinically relevant. It is also consistent with empirical findings on CBASP in outpatient settings: Schramm *et al*^16^ reported a significant difference between CBASP and non-specific psychotherapy of 2.5 points on the HDRS-24 after 20 weeks of outpatient treatment (*d*=0.31), and a meta-analysis found a significant combined overall effect of small to moderate magnitude of CBASP versus other treatments or treatment as usual (*g*=0.34–0.44).^18^

Sample size calculation was based on a simulation study.^69^ We defined a latent growth curve model (LGCM) (including latent/random intercepts and slopes) assuming that outcome changes during treatments as a function of log transformed weeks after treatment onset (ie, with larger improvements in earlier compared with later treatment phases). The focal parameter (ie, the effect of treatment groups on the latent slope) was selected to represent a three-point difference in HDRS-24 between treatments after 16 weeks. The remaining parameter values (eg, latent variances and covariances) were selected according to the results of Schramm *et al*,^16^ who shared their original HDRS-24 data with us. Given these parameter values, the expected SD of HDRS-24 scores after 16 weeks was SD=8.85, and thus the target effect of three HDRS-24 points corresponded to a standardised effect of *d*=0.34. Moreover, we assumed that each person is assessed four times across the 16-weeks interval, including a 14% dropout at the primary endpoint (assuming a linear increase of missing data across time). Given these parameters, the simulation (performed with the statistical platform R using 2000 resamples) suggested that a total sample size of n=396 yields a power of 90% to detect the target treatment effect. Simulations including effects of covariates (eg, recruitment sites) resulted in virtually identical estimates.

### Data management

All patient data collected during the course of the study in eCRFs and, in exceptional cases, in paper CRFs, will be documented with a pseudonymised identification number. The patient identification list will be kept in strict confidence at the study site in the investigator site file (ISF). The investigator at each study site is responsible for securely storing the identification list and informed consent forms.

Most of the information required by the protocol and collected during the study is entered directly into the eCRF electronically. Data are entered by patients themselves or by the investigators or a designated representative. The actimeter data are extracted from the device during study visits and transferred into the eCRF by designated study personnel (raters). Comprehensive guidelines for using the electronic data capture (EDC) system are outlined in the EDC Manual, an integral component of the ISF. The Chief Coordinator maintains a list of authorised individuals who are allowed to enter or correct data. To ensure proficient use of the EDC system, staff responsible for data entry and monitoring receive training materials and essential documentation from KKS Greifswald. All trial-related data must be accurately recorded in the eCRF by authorised individuals, as specified in the Delegation Log. User roles within the system are designed to align with the Delegation Log, ensuring compliance.

The EDC system incorporates an audit trail, enabling the traceability of documentation and database modifications at any point in time. Authorised personnel with specific access rights are permitted to make changes or corrections, with these actions being duly documented in the audit trail. Discrepancies identified during data management are communicated to monitors or the respective site promptly.

The eHealth-Platform employs personalised certificates to validate authorised access, and stored data undergo regular backups to enhance security. On completion of the study, the database will be closed following a comprehensive data cleaning process.

In a multistage process, electronically obtained data undergo thorough plausibility and consistency checks. Any identified inconsistencies, as well as missing or implausible data, are addressed through queries, and necessary corrections are implemented.

### Data analysis plan

The primary outcome will be investigated using LGCM to estimate the effect of treatment group (CBASP vs BA) on changes in HDSR-24 scores over the 16-week treatment period (T4) in the intention-to-treat (ITT) population. For the specification and estimation of the LGCM, we will use a structural equation modelling (SEM) approach with the package ‘lavaan’^70^ from the statistical environment R, or alternatively with Mplus. Compared with the model used in the power analysis, we will deviate in two respects: First, HDSR-24 scores before randomisation will be included in the model as a baseline (T0); second, the functional form of the outcome trajectories will be estimated from the data. This LGCM is equivalent to a ‘latent basis model’.^71^ The exact model specification (based on the R package ‘lavaan’) is presented in the online supplemental material 4. To account for possible deviations from a normal distribution in the primary outcome, we will use a scaled test statistic and robust standard errors. The focal parameter in this model is the effect of treatment group on the latent slope. In these and subsequent models, we will adopt a 5% significance level for testing the focal parameter and report the corresponding 95% CIs. A standardised effect size (*d*) will be computed by dividing the focal parameter from the LGCM by the pooled SD of HDRS-24 scores at 16-weeks (T4).

To include earlier data points from non-starters, treatment dropouts and study dropouts (as long as they do not withdraw consent), the LGCM will be estimated using full maximum likelihood estimation (FIML).^72^ When using FIML, missing data regarding the primary outcome variable is considered missing at random (MAR) (ie, MAR conditional on other information in the model). To make this assumption plausible, we will first correlate all available baseline variables with missingness in the primary outcome variable at later time points and then include baseline variables that predict missingness as auxiliary variables when estimating the LGCM.^73^

For sensitivity analyses, we will statistically control for the effect of selected baseline variables. These include the latent intercept of the LGCM (representing HDRS-24 scores at baseline) as well as sociodemographic and diagnostic variables for which significant differences between treatment groups occur despite randomisation. To examine whether the treatment effect depends on study sites, we will use a multiple-group LGCM (with sites representing the groups) and test whether allowing the focal parameter to vary between groups significantly improves model fit. We will also estimate the focal parameter in the per protocol (PP) population. The PP population comprises all patients who started the treatment assigned to them by randomisation and who received a therapy dosage of at least ten sessions of individual therapy and ten sessions of group therapy. Because randomisation between the two treatment groups is compromised when individuals who did not participate according to protocol are excluded, all baseline variables for which significant differences between treatment groups occur will be included as covariates in the PP analyses.

For continuous secondary and further exploratory outcomes that are measured repeatedly, we will use the LGCM with the same settings as for the primary outcome, as well as for analyses that include follow-up time points. Logistic regression analyses are planned for dichotomous outcomes such as response, remission, deterioration, relapse and dropout, which by definition cannot be measured at baseline. In all cases, focal parameters will be estimated primarily for the ITT population and additionally reported for the PP population in terms of sensitivity analyses.

All moderator and mediator analyses will be preregistered in a public repository (eg, osf.io) before running the analyses. Preregistration will include a rationale, hypothesis, variables and analytic strategy used. Briefly, for the moderator analyses, we will respecify the LGCM as a two-group model (with CBASP and BA representing the two groups) and include the hypothesised moderator variables as predictors of the latent slope. We will then test whether allowing the effects of the hypothesised moderator variables to vary between groups significantly improves model fit. In this case, we would conclude that the mean difference of latent slopes between groups (ie, the focal parameter in the respecified model) depends on the level of the baseline variables and, to that extent, they moderate the treatment effect. For the mediator analyses, we will consider all ten assessments of the primary outcome and both mediators between T0 and T4. Following recommendations by Berli *et al*,^74^ we will first look descriptively at the trajectories and correlations of these variables and then select an appropriate statistical model. For this purpose, we will consider dynamic panel models,^75^ dynamic SEM^76^ and latent growth mediation models.^77^ Sensitivity analyses, as proposed by Imai *et al.*^78^ will be conducted to determine the robustness of mediation effects to unobserved confounding variables.

Safety data will be analysed for all patients having started one of the treatments. Rates of AEs and SAEs will be calculated with corresponding two-sided 95% CIs.

### Study management

The sponsor and principal investigator (E-LB) assumes overall responsibility for this study. Along with each investigator at the study site, she bears the clinical responsibility for the research team at their respective locations.

### Data monitoring

To ensure adherence to the study protocol, compliance with relevant laws and adherence to general guidelines, comprehensive training sessions were conducted for all investigators and sub-investigators before the study’s initiation. The study was commenced by the principal investigator’s team (E-LB, SE, JPK, FP, PS, JZ) and each study site was initiated by the PI (E-LB) and a clinical monitor (AW) before enrolling the first patient. The clinical monitor stays in contact with the site coordinators on a monthly basis and visits the study centres at predefined intervals (defined by the number of enrolled study patients). Additionally, authorised representatives of the sponsor and regulatory authorities may conduct site visits to perform audits or inspections. A DSMB has been established for this study consisting of one psychologist (Professor Dr. Matthias Berking), one psychiatrist (Professor Dr. Stefan Röpke) and one biostatistician (Professor Dr. Michael Eid, 2024 replaced by Professor Dr. Steffen Nestler). It is the task of the DSMB to examine whether the conduct of the study is ethically justifiable, whether security of the patients is ensured, and whether the process of the study is acceptable. For this, the DSMB is informed about the adherence to the protocol, patient recruitment and the observed AEs.

## Ethics and dissemination

This trial adheres to the latest guidelines of the International Conference on Harmonisation-Good Clinical Practice, a globally recognised ethical and scientific standard governing the proper design, conduct, documentation and reporting of trials involving human subjects. Compliance with these standards ensures the protection of trial subjects’ rights, safety and well-being, aligning with the principles outlined in the Declaration of Helsinki.^79^ This commitment to ethical conduct also upholds the credibility of clinical trial data.

Informed consent is obtained from all participants enrolled into the study (see onlinesupplemental files 2  3 for the German and English versions). The initial version of the study protocol, patient information and consent form received approval from the Ethics Committee of University Medicine Greifswald on 04.08.2020 (registration number BB 145/20). Ethical approval has also been secured from all relevant medical Ethics Committees cognizant/responsible for the study sites. The PI will promptly notify both the DSMB and the Ethics Committee of the University Medicine Greifswald of any protocol amendments and of any events that could impact patient safety. They will also verify whether any other involved ethics committees have requested to be informed and, if so, notify them accordingly. Additionally, the Ethics Committee and the DSMB receive notifications of any SAE or SATR as well as routine or early study termination.

The study has been prospectively registered in the ClinicalTrials.gov registry (NCT04996433). We commit to publishing the study results in accordance with the Consolidated Standards of Reporting Trials (CONSORT) guidelines. Regardless of the study’s outcome, the findings will be shared in peer-reviewed journals and presented at both national and international conferences. This commitment to transparency and dissemination ensures that the knowledge gained from this trial contributes to the broader scientific community and informs future research and clinical practice.

### Patient and public involvement statement

This study prioritises active engagement of people with lived experience throughout the entire research process, starting from its initial stages. Patient involvement commenced during the inaugural pilot study of the CBASP concept conducted in Freiburg,^27^ wherein eleven patients underwent comprehensive examinations and interviews. This collaboration persisted through the second pilot study involving 70 patients^28^ and extended to other trials featuring patients from Munich^29^ and Berlin^30 31^ as well as Lübeck.^32^

The establishment of blended self-help groups in Freiburg and other cities provided a platform to incorporate the perspectives and experiences of these patients into the study’s design. Notably, a significant number of people with lived experience voiced concerns regarding the inadequacy of post-discharge care, reporting either a lack of further treatment or limited access to self-help groups, thereby increasing the risk of relapse. In response, a 6-week group therapy was integrated during the continuation phase into this study design to address this issue. Additionally, the design of this study has been significantly shaped by previously published case studies.^80^

Over the years, the researchers presenting this study have proactively shared the research framework, particularly the two psychotherapy programmes, seeking public input through numerous conferences and public outreach events. This iterative process reflects our commitment to incorporating diverse perspectives and ensuring that the study remains informed by the experiences and insights of the individuals directly affected by the research.

To conclude, the stagnation in positive findings for the treatment of depression,^81^ coupled with limited healthcare resources and the need for efficient service delivery, underscores the critical nature of our study. Although inpatient care models and admission rates vary internationally, the underlying mechanisms of PDD and the comparative effectiveness of our two treatment arms are likely to be relevant across healthcare systems. While our ChangePDD trial is conducted in Germany, its findings are expected to inform clinical decision-making and resource allocation in other countries facing similar challenges in managing PDD with TR. By providing rigorous evidence and comprehensive analysis, our trial aims to inform and influence healthcare policy and resource allocation, making it an essential endeavor in the current medical and economic climate. The potential impact on public health and policy makes the ChangePDD study not only innovative but also highly relevant and timely.

As we move forward, the findings from this trial are expected to offer substantial contributions to the field by providing evidence-based guidance for clinicians, patients and policymakers alike. We believe that our study’s unique focus and methodology will yield insights that can significantly enhance the understanding and treatment of patients suffering from PDD with TR, ultimately improving patient outcomes and the efficiency of healthcare delivery.

### Trial status

Participant enrolment began in December 2021. As of the time of publication (March 2026), 321 patients had been enrolled in the study, representing 81% of the intended sample size. The last patient entry is expected for October 2026, with data collection aimed to be completed in February 2027. Following this, an additional period of 16 months will be required to complete all follow-up assessments, data analyses and reporting, with final study completion anticipated in June 2028.

## Supplementary material

10.1136/bmjopen-2025-107051 online supplemental file 1

10.1136/bmjopen-2025-107051 online supplemental table 1

10.1136/bmjopen-2025-107051 online supplemental table 2

10.1136/bmjopen-2025-107051 online supplemental file 2

10.1136/bmjopen-2025-107051 online supplemental file 3

10.1136/bmjopen-2025-107051 online supplemental file 4
