# Supplementary material for: Efficacy, moderators and mediators of cognitive behavioural analysis system of psychotherapy (CBASP) versus behavioural activation (BA) in persistently depressed treatment-resistant inpatients: study protocol for the multicentre, randomised controlled changePDD trial
Source: BMJ Open. 2026 Apr 1;16(4):e107051. doi: 10.1136/bmjopen-2025-107051 (PMC13052729; doi:10.1136/bmjopen-2025-107051)
Supplement: online supplemental table 1 [file bmjopen-16-4-s002.pdf]

Online Supplemental Table 1.

Frequency and scope of trial visits of the ChangePDD study

	Pre-Screening	Baseline	Inpatient				Dayclinic			Continuation-Phase			Naturalistic Follow-Up					
Main Measurement Timepoints (T)	Tpre	T0	T1			T2			T3			T4						T5
Trial Visits (V)	V1a	V1b-d	V2	V3	V4	V5	V6	V7	V8	V9	V10	V11[a]	V12	V13	V14	V15	V16	V17[a]
Week	pre		1	2	4	5	6	8	10	12	14	16	24	32	40	48	56	64
Telephone screening for elegibility	x																	
Informed Consent		x																
Randomization[b]		x																
In-/Exclusion criteria		x																
Patients Master Data		x																
Patient preference		x																
ATHF / concomitant medication		x																
DIPS		x										x						x
SCID-5-PD		x										x						x
Primary Efficacy																		
HDRS-24[c]		x	**X**	x	x	**X**	x	x	**X**	x	x	**X**						x
Secondary Endpoints																		
IDS-SR		x	x	x	x		x	x	x	x	x	x	x	x	x	x	x	x
BSI, GAF[c], WHOQoL		x	x			x			x			x						x
Cost Interview		x										x						x
Further Endpoints																		
BDI-II, BRS, DAS, ECRRD8, ES, GSE, IMI-R[c], LQPT, MINI-ICF[c], MPQ, PID5BF+M, RSQ, R-GPTS, SNI, UCLA, WBI		x	x			x			x			x						x
Moderators of Change																		
CTQ, BDNF methylation[d]		x																
Mediators of Change																		
IIP-32-R, BADS, Step counts[e]		x	x	x	x		x	x	x	x	x	x						x

Add-on Study Effort Task			x			x						x		
Add-on Study EMA - IAD[f,g]		x	x	x	x	x	x	x	x	x	x	xx		
Add-on Study EMA - Social Interaction & current mood[g]		x										xx		
Side Effects /(Serious) Adverse Events														
SAEs and medication			x	x	x	x	x	x	x	x	x	x		x
SEPIPS						x			x			x		x
CSSRS[h]		x	x			x			x			x		x
Therapeutical Relationship, Adherence- and Competence Rating, Subjective Evaluation of Important Domains, Therapist														
WAI			x	x	x	x	x	x	x	x	x	x		
CBASP-CAR[c], BA-CAR[c]			x[i]											
RevieW[j]			x	x x	x	x	x x	x x	x x	x x	x x	x		
THAT[k]			x											

Note. The primary endpoint is highlighted in red.

[a] Visit 11 can be divided into smaller units and may take up to one week.

[b] Randomization will be performed between T0 and T1.

[c] Blinded observer ratings, bold capital letters indicate the primary efficacy outcome.

[d] Blood sampling for BDNF methylation will be performed at T1.

[e] Step counter will be distributed at T0 and collected at T4; for T5, step counter will be distributed again.

[f] IAD and Social interaction & mood items will be assessed four times per day for one week before admission and in week 6.

[g] IAD will be assessed once daily from week 1 to week 16.

[h] CSSRS may be used voluntarily at any other visit if deemed necessary.

[i] One rating per patient will be randomly selected from a pool of at least six videos per patient.

[j] Two crosses per cell are used because the questionnaire Review is conducted weekly.

[k] THAT will be completed by each active study therapist once certified.

General abbreviations: **BA** = Behavioral Activation, **BDNF** = Brain-derived neurotrophic factor, **CBASP** = Cognitive Behavioral Analysis System of Psychotherapy, **DSM** = Diagnostic and Statistical Manual of Mental Disorders, **T** = Measurement Time Point, **SAE** = Serious Adverse Events, **V** = Visits.

Abbreviations of all instruments with their main reference: **ATHF** = Antidepressant Treatment History Form[1], **BA-CAR:** Observer based Competence and Adherence Rating of BA (based on the Cognitive Therapy Scale, CTS[2]), **BADS** = Behavioral Activation of Depression Scale[3], **BDI-II** = Beck's Depression Inventory[4], **BRS** = Brief Resilience Scale[5], **BSI** = Brief Symptom Inventory[6], **CBASP-CAR** = Observer based Competence and Adherence Rating of CBASP (based on the Cognitive Therapy Scale, CTS[2]), **CTQ** = Childhood Trauma Questionnaire[7-9], **Cost Interview**[10], **DAS** = Dysfunctional Attitude Scale[11], **ECR-RD8** = Short version of the Experiences in Close Relationships - Revised questionnaire[12], **ES** = Euthymia Scale[13], **GAF** = Global Assessment of Functioning[14], **GSE** = General Self-Efficacy Scale[15], **HDRS-24** = Hamilton Depression Rating Scale, 24-item version[16,17], **IAD** = Interpersonal-Activation-Diary[18], **IDS-SR** = Inventory of Depressive Symptoms, Self Report[19], **IIP-32** = Inventory of Interpersonal Problems Short

Form[20], **IMI-R** = Impact Message Inventory revised[21], **LQPT** = Lübecker Questionnaire of Preoperational Thinking[22], **MINI-ICF** = Measure of disorders of capacity as defined by the International Classification of Functioning[23], **MPQ** = Mental Pain Questionnaire[24], **PID5BF+M** = Personality Inventory for DSM-5 Brief Form Plus – Modified[25], **RevieW** = Review of the last Week concerning helpful domains for change[26], **R-GPTS** = Revised-green paranoid thoughts scale[27], **RSQ** = Rejection Sensitivity Questionnaire[28], **SCID-5-PD** = Structured Clinical Interview for DSM-5 – Personality Disorders[29], **SEPIPS** = Side Effects of Psychological Interventions Process Scale[30], **SNI** = Social Network Index[31], **THAT** = Questionnaire concerning Psychotherapeutic Identity and Attitude[32], **UCLA** = UCLA Loneliness Scale[33], **WAI** = Working Alliance Inventory[34], **WBI** = Well-Being Index[35], **WHOQoL** = World Health Organization Quality of Life[36].

References:

1. Sackeim HA, Aaronson ST, Bunker MT, Conway CR, Demitrack MA, George MS, et al. The assessment of resistance to antidepressant treatment: rationale for the Antidepressant Treatment History Form—Short Form (ATHF-SF). *J Psychiatr Res* 2019;113:125–36. doi:10.1016/j.jpsychires.2019.03.021
2. Weck F, Hautzinger M, Heidenreich T, Stangier U. Erfassung psychotherapeutischer Kompetenzen: Validierung einer deutschsprachigen Version der Cognitive Therapy Scale. *Z Klin Psychol Psychother* 2010;39:244–50. doi:10.1026/1616-3443/a000055
3. Kanter JW, Mulick PS, Busch AM, Berlin KS, Martell CR. The Behavioral Activation for Depression Scale (BADS): psychometric properties and factor structure. *J Psychopathol Behav Assess* 2007;29:191–202. doi:10.1007/s10862-006-9038-5
4. Hautzinger M, Keller F, Kühner C. *Beck Depression Inventar II (BDI-II).* Frankfurt am Main: Harcourt Test Services; 2006.
5. Smith BW, Dalen J, Wiggins K, Tooley E, Christopher P, Bernard J. The Brief Resilience Scale: assessing the ability to bounce back. *Int J Behav Med* 2008;15:194–200. doi:10.1080/10705500802222972
6. Derogatis LR, Spencer PM. *Brief Symptom Inventory (BSI).* Upper Saddle River (NJ): Pearson; 1993.
7. Bernstein DP, Fink LA. *Childhood Trauma Questionnaire: a retrospective self-report.* San Antonio (TX): Psychological Corporation; 1998.
8. Bernstein DP, Stein JA, Newcomb MD, Walker E, Pogge D, Ahluvalia T, et al. Development and validation of a brief screening version of the Childhood Trauma Questionnaire. *Child Abuse Negl* 2003;27:169–90.
9. Wingenfeld K, Spitzer C, Mensebach C, Grabe HJ, Hill A, Gast U, et al. The German version of the Childhood Trauma Questionnaire (CTQ): preliminary psychometric properties. *Psychother Psychosom Med Psychol* 2010;60:442–50.
10. Wagner T, Fydrich T, Stiglmayr C, Marschall P, Salize HJ, Renneberg B, et al. Societal cost-of-illness in patients with borderline personality disorder before, during and after dialectical behaviour therapy. *Behav Res Ther* 2014;61:12–22. doi:10.1016/j.brat.2014.07.004
11. Hautzinger M, Joormann J, Keller F. *Dysfunktionale Einstellungen: DAS – Manual.* Göttingen: Hogrefe; 2005.
12. Ehrenthal JC, Zimmermann J, Brenk-Franz K, Dinger U, Schauenburg H, Brähler E, et al. Evaluation of a short version of the Experiences in Close Relationships-Revised questionnaire (ECR-RD8). *BMC Psychol* 2021;9:140. doi:10.1186/s40359-021-00637-z
13. Carrozzino D, Svicher A, Patierno C, Berrocal C, Cosci F. The Euthymia Scale: a clinimetric analysis. *Psychother Psychosom* 2019;88:119–21. doi:10.1159/000496230
14. Hall RC. Global assessment of functioning: a modified scale. *Psychosomatics* 1995;36:267–75.
15. Jerusalem M, Schwarzer R. *Skala zur Allgemeinen Selbstwirksamkeitserwartung (SWE).* 2003. Available: https://www.psycharchives.org/en/item/d041ef33-7cb7-4b29-a92d-29d900fc462b.

16. Hamilton M. A rating scale for depression. *J Neurol Neurosurg Psychiatry* 1960;23:56–62. doi:10.1136/jnnp.23.1.56
17. Williams JB. A structured interview guide for the Hamilton Depression Rating Scale. *Arch Gen Psychiatry* 1988;45:742–7. doi:10.1001/archpsyc.1988.01800320058007
18. Zimmermann, J., Woods, W. C., Ritter, S., Happel, M., Masuhr, O., Jaeger, U., Spitzer, C., & Wright, A. (2019). Integrating structure and dynamics in personality assessment: First steps toward the development and validation of a personality dynamics diary. Psychological assessment, 31(4), 516–531. doi.org/10.1037/pas0000625
19. Rush AJ, Carmody T, Reimitz PE. The Inventory of Depressive Symptomatology (IDS): clinician and self-report ratings. *Int J Methods Psychiatr Res* 2000;9:45–59.
20. Horowitz LM, Alden LE, Kordy H, Strauß B. *Inventar zur Erfassung interpersonaler Probleme (IIP-D).* Weinheim: Beltz Test; 2000.
21. Casper F, Fingerle H, Werner M. *Der Impact Message Inventory (IMI): ein Instrument zur interpersonalen Fremdbeurteilung.* Freiburg: Universität Freiburg; 2000.
22. Kühnen T, Knappke F, Otto T, Friedrich S, Klein JP, Kahl KG, et al. Chronic depression: development and evaluation of the Lübeck Questionnaire for Recording Preoperational Thinking (LQPT). *BMC Psychiatry* 2011;11:199. doi:10.1186/1471-244X-11-199
23. Linden M, Baron S. The Mini-ICF rating for mental disorders (Mini-ICF-P). *Rehabilitation (Stuttg)* 2005;44:144–51. doi:10.1055/s-2004-834786
24. Fava GA. Well-being therapy: current indications and emerging perspectives. *Psychother Psychosom* 2016;85:136–45. doi:10.1159/000444114
25. Bach B, Kerber A, Aluja A, Bastiaens T, Keeley JW, Claes L, et al. International assessment of DSM-5 and ICD-11 personality disorder traits. *Psychopathology* 2020;53:179–88. doi:10.1159/000507589
26. Koy J. *Specific and non-specific working mechanisms of inpatient psychotherapy in depression.* Master's thesis. Marburg: Philipps-Universität Marburg; 2019.
27. Freeman D, Loe BS, Kingdon D, Startup H, Molodynski A, Rosebrock L, et al. The revised Green et al. Paranoid Thoughts Scale (R-GPTS). *Psychol Med* 2021;51:244–53. doi:10.1017/S0033291719003155
28. Downey G, Feldman SI. Implications of rejection sensitivity for intimate relationships. *J Pers Soc Psychol* 1996;70:1327–43. doi:10.1037/0022-3514.70.6.1327
29. First MB, Williams JBW, Benjamin LS, Spitzer RL. *Structured Clinical Interview for DSM-5 Personality Disorders (SCID-5-PD).* Arlington (VA): American Psychiatric Association Publishing; 2016.
30. Herzog P, Kaiser T, Gärtner T, Rief W, Brakemeier E-L. Detecting and monitoring side effects of psychological interventions: development and validation of the SEPIPS scale. *Manuscript in preparation*.
31. Cohen S, Doyle WJ, Skoner DP, Rabin BS, Gwaltney JM. Social ties and susceptibility to the common cold. *JAMA* 1997;277:1940–4. doi:10.1001/jama.1997.03540480040036
32. Klug G, Huber D, Kächele H. *Psychotherapeutische Haltung (THAT).* Stuttgart; 2002.
33. Russell D, Peplau LA, Cutrona CE. The revised UCLA Loneliness Scale. *J Pers Soc Psychol* 1980;39:472–80. doi:10.1037/0022-3514.39.3.472
34. Wilmers F, Munder T, Leonhart R, Herzog T, Plassmann R, Barth J, et al. Working Alliance Inventory – Short Revised (WAI-SR), German version. Göttingen: Vandenhoeck & Ruprecht; 2008.
35. Krieger T, Zimmermann J, Huffziger S, et al. Measuring depression with the WHO-5 Well-Being Index. *J Affect Disord* 2014;156:240–4. doi:10.1016/j.jad.2013.12.015
36. Angermeyer MC, Kilian R, Matschinger H. *WHOQOL-100 und WHOQOL-BREF.* Göttingen: Hogrefe; 2000.
