# Supplementary material for: Efficacy, moderators and mediators of cognitive behavioural analysis system of psychotherapy (CBASP) versus behavioural activation (BA) in persistently depressed treatment-resistant inpatients: study protocol for the multicentre, randomised controlled changePDD trial
Source: BMJ Open. 2026 Apr 1;16(4):e107051. doi: 10.1136/bmjopen-2025-107051 (PMC13052729; doi:10.1136/bmjopen-2025-107051)
Supplement: online supplemental table 2 [file bmjopen-16-4-s003.pdf]

Online Supplemental Table 2.

Characteristics and definitions of primary, safety, secondary, and exploratory endpoints of the ChangePDD study

Outcome	Instrument	Rating	Domain	Level of measurement
**Primary**	HDRS-24	Clinician-rated	Change in depression severity after 16 weeks	Continuous
**Safety**	AEs and SAEs	Documentation of study sides	(Serious) adverse events	Dichotomous
**Safety**	SEPIPS	Self-rated	Side effects	Continuous
**Secondary**	HDRS-24	Clinician-rated	Rates of response (>50% reduction from baseline), remission (HDRS score <10 post-treatment) and deterioration (HDRS score at least 6 points increase from baseline)	Dichotomous
**Secondary**	Relapse	Clinician-rated	Defined as rehospitalization for symptomatic worsening and/or a combination of an increase in HDRS-24 from discharge of equal or greater than 10 points and a current HDRS-24 score of equal or greater than 18 points at T5	Dichotomous
**Secondary**	Treatment dropout rate	Documentation of study sides	Discontinuation of the study treatment before T4 and T5	Dichotomous
**Secondary**	Cost Interview	Clinician-rated	Societal cost of illness	Continuous
**Secondary**	GAF	Clinician-rated	Global functioning	Continuous
**Secondary**	BSI	Self-rated	General distress	Continuous
**Secondary**	IDS-SR	Self-rated	Depressive symptoms	Continuous
**Secondary**	WHOQoL	Self-rated	Quality of life	Continuous
**Moderator**	CTQ	Self-rated	Childhood maltreatment	Continuous
**Moderator**	BDNF methylation	Laboratory values	Epigenetic mechanism	Continuous
**Mediator**	IIP-32-R	Self-rated	Interpersonal problems	Continuous
**Mediator**	BADS	Self-rated	Behavioral activation	Continuous
**Exploratory**	ATHF/ medication	Clinician-rated	Medication and antidepressant use	Continuous
**Exploratory**	CSSRS	Clinician-rated	Suicidal thoughts	Continuous
**Exploratory**	DIPS	Clinician-rated	Clinical diagnostics	Continuous
**Exploratory**	IMI-R	Clinician-rated	Interpersonal impact	Continuous
**Exploratory**	MINI-ICF	Clinician-rated	Global functioning	Continuous
**Exploratory**	SCID-5-PD	Clinician-rated	Clinical diagnostics	Continuous
**Exploratory**	BDI-II	Self-rated	Severity of depression	Continuous
**Exploratory**	BRS	Self-rated	Resilience	Continuous
**Exploratory**	DAS	Self-rated	Dysfunctional attitudes	Continuous
**Exploratory**	ECR-RD8	Self-rated	Attachment styles	Continuous
**Exploratory**	ES	Self-rated	Euthymia	Continuous
**Exploratory**	GSE	Self-rated	Self-Efficacy	Continuous
**Exploratory**	LQPT	Self-rated	Preoperational thinking	Continuous
**Exploratory**	MPQ	Self-rated	Mental pain	Continuous
**Exploratory**	PID5BF+M	Self-rated	Maladaptive personality traits	Continuous
**Exploratory**	RevieW	Self-rated	Therapeutic progress	Continuous

**Exploratory**	RSQ	Self-rated	Rejection sensitivity	Continuous
**Exploratory**	R-GPTS	Self-rated	Paranoia	Continuous
**Exploratory**	SNI	Self-rated	Interpersonal / social network	Continuous
**Exploratory**	UCLA-LS	Self-rated	Loneliness	Continuous
**Exploratory**	WBI	Self-rated	Well-being	Continuous
**Exploratory**	WAI	Self-rated and therapist-rated	Working alliance, therapeutic relationship	Continuous
**Exploratory**	Step count	Actimeter	Steps	Continuous

Note. Abbreviations and corresponding references for all instruments are provided in the note accompanying Online Supplemental Table 1.
