# Supplementary material for: Efficacy, moderators and mediators of cognitive behavioural analysis system of psychotherapy (CBASP) versus behavioural activation (BA) in persistently depressed treatment-resistant inpatients: study protocol for the multicentre, randomised controlled changePDD trial
Source: BMJ Open. 2026 Apr 1;16(4):e107051. doi: 10.1136/bmjopen-2025-107051 (PMC13052729; doi:10.1136/bmjopen-2025-107051)
Supplement: online supplemental file 2 [file bmjopen-16-4-s004.pdf]

Patient Information Sheet

**Study title:**	**A Comparison of Two Different Psychotherapy Programs in Persistently Depressed Treatment-Resistant Inpatients**
**Short title:**	**ChangePDD**
**Responsible study physician** (investigating physician)	Name
**Study center:**	Name and address of the specific study site
**Study leadership**	Prof. Dr. Eva-Lotta Brakemeier<br>Institut für Psychologie<br>Franz-Mehring-Straße 47<br>17489 Greifswald<br>Telefon: +49-3834-420-3718 / Fax: +49-3834-420-3763<br>E-Mail: eva-lotta.brakemeier@uni-greifswald.de
**Data protection officer of the study site**	
**Data protection supervisory authority of the federal state in which your study site is located**	
**Data protection officer of the study leadership**	

Dear Patient,

We would like to ask whether you are willing to participate in the clinical study described below. The study acronym ***ChangePDD*** reflects our goal: Through this study, we aim to change long-lasting depression (persistent depressive disorder = PDD, most often referred to as chronic depression in Germany) using psychotherapeutic programs.

Clinical studies are necessary to gain knowledge about the effectiveness of treatment methods or to expand existing knowledge.

The clinical study we are presenting to you has been reviewed by an ethics committee. The ethics committee has raised no ethical concerns.

This clinical study is being conducted at study centers across several locations in Germany. A total of 396 patients are expected to participate.

The study is coordinated under the leadership of Prof. Dr. Eva-Lotta Brakemeier at the Institute of Psychology, University of Greifswald, and is funded by the German Research Foundation (DFG).

Your participation in this clinical study is voluntary. You will only be included in the study if you provide your written consent to participate and to the processing of your personal data. You may withdraw your consent at any time, with future effect, without giving any reasons and without experiencing any disadvantages as a result.

You have already been informed about the planned study. The following information is intended to explain the study's objectives and procedures. In addition, a therapist will conduct an informed-consent discussion with you, during which they will explain all points mentioned here once again. Please do not hesitate to address anything that is unclear to you. You will then be given sufficient time to decide whether you wish to participate.

Brief Description of the Study

Reason for the study: You are affected by a long-lasting or chronic depression and have not responded sufficiently to previous treatment attempts. This study aims to compare the effectiveness of a newer psychotherapy specifically developed for chronic depression (called "CBASP") with an already established psychotherapy (called "BA").
CBASP is a relatively new psychotherapy developed for patients with chronic depression and difficult relationship experiences in childhood.
The already well-established psychotherapy BA is a variant of cognitive behavioral therapy and has been shown in numerous studies to be effective in treating depression.
These two therapeutic approaches have never been compared directly, so we do not yet know which treatment program is more effective for chronic depression.

Study procedure: If you decide to participate, the study will last a total of 16 months (64 weeks) for you. During the first 4 months (16 weeks), you will receive intensive treatment in a partially inpatient, day-clinic, or outpatient setting. Twelve months later, you will be invited for a follow-up assessment.

Possible benefits for you: Both therapies are known to have a positive effect on chronic depression. By participating, you also make a valuable scientific and health-policy contribution to improving inpatient psychotherapy programs in both the short and long term.

Risks and burdens: In any psychotherapy, temporary distress may occur due to actively addressing topics discussed in therapy sessions that you may previously have avoided. Filling out questionnaires and completing interviews throughout the study (especially at the beginning) may also be burdensome for you.
Please inform us immediately of any deterioration in your health, regardless of whether you believe it is related to the study.

Voluntariness: It is entirely your decision whether or not you wish to participate in this study. You may say NO at any time—either immediately or later. You do not have to provide any reasons, and you will not experience any disadvantages.
In addition to the written information, you will receive an oral explanation. Please ask the therapist if anything is unclear to you. You will then have sufficient time to decide whether to participate. If you decide to take part, please sign the consent form.

Table of Contents

PART I: INFORMATION ON THE COURSE OF THE CLINICAL TRIAL AND HEALTH-RELATED ASPECTS 5

I. 1. Why is this study being conducted? 5

I. 1. 1. Will I definitely receive treatment? 6

I. 1. 2. What is the course of the study and what should I know as a participant? What data are collected during the study? 6

I. 2. What are the benefits of participating in the study? 9

I. 3. What risks are associated with participation in the study? 9

I. 4. What other treatment options are available? 10

I. 5. Who is not eligible to participate in this clinical study? 11

I. 6. Will participation in the study incur any additional costs for me? Is compensation provided? 11

I. 7. Am I insured during the study? 11

I. 8. Who can I contact during the study? 11

PARTL II: INFORMATION ON DATA PROTECTION AND BIOLOGICAL SAMPLES 12

II. 1. What happens to my personal data? 12

a) General Information 12
b) Legal Basis 12
c) Responsibility 12
d) Purpose 12
e) Disclosure/Recipients 12
f) Your Rights under the DSGVO (Datenschutz-Grundverordnung) 13

II. 2. What happens to my biological samples? 15

a) Use of your biological samples 15
b) Storage 15
c) Discolsure/Recipients 15
d) Your rights under the DSGVO (Datenschutz-Grundverordnung) 15

CONSENT FORM 17

Part I: Information on the Course of the Clinical Trial and Health-Related Aspects

I. 1. Why is this study being conducted?

Depressive disorders follow a long-lasting or chronic course in about one third of all cases, despite attempts at treatment with medication and/or psychotherapy. These depressions are also referred to as chronic or persistent depression. The criterion for this diagnosis is that the depression lasts longer than two years.

The aim of the study is to compare, in patients with long-lasting or chronic depression who have not responded sufficiently to previous treatment attempts, the effectiveness of a newer psychotherapy specifically developed for chronic depression (called “CBASP”) with that of an already established psychotherapy (called “BA”). What exactly are CBASP and BA?

The somewhat cumbersome name **CBASP** stands for “**Cognitive Behavioral System of Psychotherapy**.” The long title reflects the fact that strategies from different psychotherapeutic traditions are integrated. CBASP is a relatively new psychotherapy developed for patients with chronic depression and difficult relationship experiences in childhood. Its effectiveness has already been demonstrated in outpatient studies. In CBASP, both childhood experiences and current problems are addressed using relatively structured methods.

BA stands for “**Behavioral Activation**.” This well-established psychotherapy is a variant of cognitive behavioral therapy and has been shown in numerous studies to be effective in treating depression. BA considers activation to be a decisive factor in depression treatment. Activation is most successful when activities are derived from the patient’s values (that is, life themes and life goals), which is why values are explored during therapy.

These two therapeutic approaches have not yet been directly compared, so we do not know which treatment program is more effective for chronic depression. Both psychotherapy programs are first delivered in an inpatient setting—which may be supplemented by a day-clinic phase, depending on your clinic’s structure—and then as outpatient group therapy. The number of therapy sessions does not differ; only the content of the therapy programs varies.

By conducting this clinical study, we hope to tailor the treatment of patients with chronic depression even more effectively to their individual needs and to expand our knowledge about how the two therapy programs, CBASP and BA, work.

I. 1. 1. Will I definitely receive treatment?

If you choose to participate, you will definitely receive one of the two therapy programs. Which treatment you receive is determined by chance (this process is called randomization). The probability of receiving CBASP or BA is 50% each. You will be informed immediately after randomization which therapy you will receive.

I. 1. 2. What is the course of the study and what should I know as a participant? What data are collected during the study?

By participating in the study, you will receive a psychotherapeutic treatment program. The treatment will be conducted by a therapy team trained in psychotherapy.

You will be treated for a total of 16 weeks: first 5 weeks as an inpatient, followed by 5 weeks in a day-clinic/partial inpatient setting, and then 6 additional weeks of outpatient therapy. Twelve months later, you will be invited for a follow-up assessment. The total duration of your participation in the study is therefore 16 months (i.e., 64 weeks).

During the 5-week inpatient phase and the 5-week day-clinic phase, you will receive per week:

- 2 individual therapy sessions (duration: 50 minutes each)
- 2 group therapy sessions (duration: 100 minutes each)
- 1 primary nursing session (therapeutic exchange with a nurse) (duration: 25 minutes)
- 1 movement therapy session (duration: 75 minutes)

During the 6-week outpatient treatment phase, you will receive per week:

- 1 group therapy session (duration: 100 minutes)

This therapy program is similar in number and type of therapies to routine treatment, but it is somewhat more structured and longer:

- More structured, because treatment starts inpatient, then continues day-clinic, and finally outpatient.
- Longer, because outpatient group therapy is not usually offered to all patients. In this study, your treatment will last 4 months (16 weeks).

In addition, you may participate in the regular therapy programs offered on the ward (such as occupational therapy and physiotherapy) and may receive medication according to a treatment plan based on current clinical guidelines for depression. To obtain accurate conclusions about the effectiveness of your therapy within the ChangePDD study, it is unfortunately not possible to participate in all available therapy programs. **Please discuss with your therapist which programs you can use locally.**

In addition to your therapeutic treatment, study visits will take place at regular intervals. Table 1 (see below) illustrates the types of visits as well as the timeline across the entire study. As shown in the table, there are six main assessment points, which occur before the inpatient admission: the baseline assessment (T0), at the start of treatment (T1), after 5 weeks of inpatient treatment (T2), at the end of the inpatient or day-clinic phase (T3), at the end of the outpatient group therapy (T4), and at the 12-month follow-up, i.e., the final assessment (T5).

In addition, the first column of Table 1 lists the various study visits, which are described in more detail below:

- **Baseline data and blood sample:** Upon inpatient admission, we will collect your general demographic information such as age, gender, and relationship status (baseline data). We will also take approximately 10 ml of blood to measure a specific biomarker in your blood (the so-called BDNF methylation). This biomarker reflects biological processes and may serve as an indicator for environmental stress and depression. It will be analyzed to determine what role this biomarker may play in the effectiveness of psychotherapeutic methods in the treatment of depression.

- **Long and short clinical interviews:** During the interviews, a member of the study team—who is not informed about which of the two psychotherapy programs you are receiving—will ask you about your well-being. As part of the so-called “cost interviews,” you will also be asked questions about your physical and mental health and the related use of various healthcare services, as well as your employment situation and living situation. This allows us to relate treatment costs, sick days, etc. to the benefits of the psychotherapy programs. The long interviews will last approximately 90 to 120 minutes, and the short ones about 15 to 20 minutes. Breaks can of course be taken at any time.

- **Long and short questionnaire assessments:** During these visits, you will be asked to complete questionnaires covering various areas (e.g., depression, other symptoms, well-being, different areas of life, childhood). The long questionnaire assessment is more extensive than routine diagnostics. You will complete up to 23 questionnaires, which will take between 60 and 90 minutes. Eight of these questionnaires are also part of routine treatment, so your maximum additional time burden will be about 60 minutes at certain assessment points. Breaks may of course be taken at any time. During the short assessments, you will complete only a few questionnaires, taking about 10 to 15 minutes.

- **Questions about side effects:** Since psychotherapy—like any treatment—may also cause side effects, we consider it important to ask about them. Completing the related questionnaires will take 10–15 minutes.

- **Brief evaluation of the treatment:** At the end of each treatment week, we will ask you to indicate which strategies you applied during the past week and how they affected your depressive symptoms. Answering these questions will take 10–15 minutes.

- **Step counter:** For the duration of the psychotherapy program (16 weeks), you will receive a step counter in the form of a wristwatch. We ask you to wear it continuously for all 16 weeks so that your steps are automatically recorded (see continuous arrow in Table 1). You will receive the step counter once again one week before the final assessment, so we can measure your steps at that time as well. This enables us to examine whether there is a connection between your physical activity and the course of your depression.

- **Unscheduled visits:** Not everything always goes as planned. If more than three months pass between your baseline assessment and your inpatient admission, we will ask you to repeat some questionnaires and interviews (see "Long and short questionnaire assessments") in an additional visit.

Data collection at the beginning of the study (T0) is particularly extensive and therefore takes place over several days. For comparability, we will collect data to the same extent at time points T4 and T5. At these time points as well, you may divide the data collection across several days during unscheduled visits.

Table 1: Overview of the Type and Timeline of Study Visits

	Screening	Inpatient treatment				Day clinic treatment			Outpatient treatment			Follow-up observation					
Measurement time point	T0	T1			T2			T3			T4						T5
Week		1	2	4	5	6	8	10	12	14	16	24	32	40	48	56	64
Baseline data	X																
Blood sampling		X															
Long clinical interview	X	X			X			X			X						X
Short clinical interview			X	X		X	X		X	X							
Long questionnaire assessment	X	X			X			X			X						X
Short questionnaire assessment			X	X		X	X		X	X		X	X	X	X	X	
Adverse effects questionnaire					X			X			X						
Brief treatment evaluation		X	XX	X	X	XX	XX	X	XX	XX	XX						X
Step counter		X	X	X	X	X	X	X	X	X	X						X

Strict adherence to the scheduled study visits and therapy sessions is crucial for the success of the study. During your participation in the ChangePDD study, you may not take part in another study unless it is observational (non-interventional) in nature. If you wish to participate in another study, please discuss this in advance with your responsible therapist. During the 16 weeks of the psychotherapy programs, any additional medication or other psychotherapeutic treatments should only be undertaken in consultation with the study therapists.

I. 2. What are the benefits of participating in the study?

By participating, you make a valuable scientific and health-policy contribution to improving inpatient psychotherapy programs in both the short and long term.

Possible advantages and opportunities for you:

- No long waiting period for admission to the clinic,
- Comprehensive and valuable assessment of your problems throughout the entire treatment,
- Intensive psychotherapeutic (partial) inpatient treatment for depression,
- Participation in outpatient group therapy after discharge to maintain your progress and prevent relapse,
- Inclusion in a larger scientific study with close support from trained staff who are always available to assist you.

There is a high likelihood that both intensive psychotherapy programs (CBASP and BA) will have a positive impact on your chronic depression and help improve your problems. However, it is also possible that you may not experience any direct benefit to your mental health from participation. The overall results of this scientific investigation aim to help identify effective treatments for the group of patients with chronic depression and to increase the likelihood that each patient can be matched with the therapy approach that is best suited for them individually.

I. 3. What risks are associated with participation in the study?

In any psychotherapy, temporary distress may occur from actively addressing topics discussed in therapy sessions that you may have previously avoided. This can, in some cases, lead to the emergence or increase of suicidal thoughts or plans. You will be asked regularly about suicidality; additionally, we urge you to contact study staff or appropriate emergency services immediately if you experience suicidal thoughts.

Completing questionnaires and participating in interviews throughout the study—especially at the beginning—may also be stressful.

Please inform us immediately of any deterioration in your health, regardless of whether you believe it is related to the study. During the inpatient phase, you can consult your therapists at any time, and during the outpatient phase, you can contact study staff (see contact details at the end of this information). If the burden becomes too great, you may also stop completing questionnaires, participating in interviews, or withdraw from the study at any time without any disadvantage. In case of withdrawal, you will continue to receive clinical care as any non-study patient.

As part of the study, a single blood sample (approximately 10 ml) will also be taken. Blood draws are generally associated with very low risk. You may experience mild pain at the puncture site or develop a bruise that may be visible for a few days. In very rare cases, a blood clot (thrombosis), localized inflammation, infection, or permanent damage to blood vessels or nerves at the puncture site can occur.

I. 4. What other treatment options are available?

If you do not participate in the study, the usual treatments at the clinic are available to you. Please ask your therapist if you want to know which alternative treatments are available should you choose not to participate in the study.

I. 5. Who is not eligible to participate in this clinical study?

You may not participate in this clinical study if you have already received CBASP or BA therapy within the past year, or if this type of therapy is not suitable for you.

I. 6. Will participation in the study incur any additional costs for me? Is compensation provided?

Participation in this study does not incur any additional costs for you. Accordingly, no financial compensation will be paid for participation in this study. Co-payments for patients with statutory health insurance according to §39 (4) SGB V may still apply and are unaffected by study participation.

I. 7. Am I insured during the study?

This clinical study focuses on the two psychotherapy methods, CBASP and BA. No medications or medical devices are administered. Therefore, no special clinical trial insurance is required.

However, we have taken out travel accident insurance for you, which covers accidents that may occur on your way to or from study participation.

Insurance provider:

Insurance number:

If you suspect that your health has been harmed or pre-existing conditions worsened as a result of participating in the study, you must report this immediately to your therapist.

I. 8. Who can I contact during the study?

You always have the opportunity for further consultation with your therapist or the study team listed on page 1 to clarify any questions related to the study. Please do not hesitate to contact your therapist or the study team if you have any questions. Questions regarding your rights and responsibilities as a patient and study participant will also be gladly answered.

Partl II: Information on Data Protection and Biological Samples

II. 1. What happens to my personal data?

a) General Information
Within the framework of this study, both psychological-medical findings (e.g., current examination results, medications) and demographic data (age, gender) will be processed in a pseudonymized form. "**Pseudonymized**" means that your personal data are assigned an artificial identifier (consisting of letters and numbers) so that no direct connection to your person is possible. A connection between the data and your identity is only possible via an identification list stored with your therapist. Your data are protected against unauthorized access.

The storage and retention of personal data take place in pseudonymized form at your study center and with the responsible study director, Prof. Dr. Eva-Lotta Brakemeier (University of Greifswald).

After the study ends, all pseudonymized data will be stored and retained according to the regulations valid at that time. These pseudonymized data are accessible only to study staff. Currently, the legally required retention period for study documents is 10 years (§13 para. 10 GCP-V).mFurthermore, after the conclusion of the study, personal data will be processed in **anonymized** form for further scientific research and publications. "**Anonymized**" means that the data can no longer be attributed to you as a person after the pseudonyms are deleted.

b) Legal Basis
The legal basis for your participation in the study and the processing of your personal data is your informed consent in accordance with the EU General Data Protection Regulation (Art. 6 para. 1 lit. a in conjunction with Art. 9 para. 2 lit. a GDPR). Without your explicit consent to the processing of your personal data, participation in this study is unfortunately not possible.

c) Responsibility
The entity responsible under the General Data Protection Regulation is:
University of Greifswald
Represented by

d) Purpose
The purpose of data processing is solely the planning, execution, and evaluation of the ChangePDD study. Further information on the study can be found in the patient information sheet.

e) Disclosure/Recipients
Only study staff have access to your data. These individuals are bound by confidentiality and data protection. The data are protected against external access. All personal data will be transmitted pseudonymized from your study center to Prof. Eva-Lotta Brakemeier (University of Greifswald, head of the clinical study).

The personal data you provide as part of the above-mentioned clinical study (including the original clear data) may, if required and legally permitted, be inspected by the competent

supervisory authority during inspections or by representatives of the study management (so-called auditors or monitors) to verify the proper conduct of the clinical trial at the study site. These individuals are obligated to maintain confidentiality, and personal data will not be further shared in this context.

If you give separate consent to inform your primary care physician/psychiatrist/neurologist, they will be informed about your participation in the study.

There is no transfer of personal data to a third country or to an international organization.

f) Your Rights under the DSGVO (Datenschutz-Grundverordnung)
You generally have the following rights regarding your personal data, unless it is technically impossible or prohibited by law due to the deletion of identifying features (pseudonyms) for decryption:

Right to withdraw your consent
Just like your consent to participate in the clinical study, you can also withdraw your consent to the processing of your personal data at any time without giving reasons, with effect for the future. The legality of the data processing carried out until the withdrawal remains unaffected.

Both your consent to participate in the study and your consent to data processing are prerequisites for your participation in this study.

You also have the following rights:
You have the right to request free information about your stored personal data, their origin and recipients, the purpose, and the duration of data processing. In addition, under certain conditions, you have the right to request correction, restriction of processing (e.g., blocking), deletion, and data portability.

Furthermore, you have the right to lodge a complaint with the competent data protection supervisory authority if you believe that the processing of your personal data is unlawful. The competent supervisory authority is listed on the cover sheet of the patient information under the section “Data Protection Supervisory Authority” for the federal state in which your study site is located.

A list of all data protection supervisory authorities in Germany and the European Union can be found here:
https://www.bfdi.bund.de/DE/Infothek/Anschriften_Links/anschriften_links-node.html

Exercising your rights
If you wish to exercise one or more of the rights mentioned, please contact your study physician.

Please note that the exercise of your rights is only possible until the data are anonymized, since afterwards the anonymized data can no longer be attributed to any person.

For concerns regarding the processing of personal data and compliance with data protection requirements, you can also contact the data protection officer of the study site or the study management listed on the cover sheet of the patient information.

You also have the right to contact the study management directly at any time. However, in general, please contact your study physician or the data protection officer of your study site, since due to pseudonymization, your identity is only known at your treating center, which allows further steps to be taken appropriately and avoids inadvertent identification by the study management

.

II. 2. What happens to my biological samples?

During this clinical study, biological samples (blood samples) will be collected from you in order to determine a specific biomarker in the blood (the so-called BDNF methylation). The samples will be stored in pseudonymized form, just like your other personal data (for pseudonymization, see above II.1.a).

However, the pseudonymization of your biological samples does not necessarily offer the same level of protection as the pseudonymization of personal data. Your biological samples always contain information about your genetic material, which could potentially be used for identification. In addition, your samples contain sensitive genetic information, which could reveal details about family relationships or hereditary diseases. The risk of identification cannot be completely excluded and increases the more data can be linked together—for example, if you publish genetic data online (e.g., for ancestry research).

Since personal information can be derived from your biological samples, the data protection provisions mentioned above under II.1 also apply to your biological samples.

a) Use of your biological samples
The biological samples allow biological processes to be measured and may serve as an indicator of environmental exposures and depression. This information will be analyzed to determine the role of this biomarker in the effectiveness of psychotherapeutic methods for treating depression.

Preliminary study results already suggest that this biomarker could be relevant for treatment response. Based on these results, we plan to conduct genome-wide association studies and candidate gene studies (studies designed to link this potential biomarker to specific courses of depression). In the future, this could make it possible to use computer-based algorithms to assign patients to the therapy that best matches their individual needs based on this biomarker.

b) Storage
The samples will be stored in the biobank of the Hannover Medical School. The biological samples will be kept in pseudonymized form for 5 years in accordance with the guidelines of Hannover Medical School and then anonymized (for anonymization, see above II.1.a).

c) Discolsure/Recipients
The blood samples will only be processed in pseudonymized form for this clinical study and sent to the Laboratory for Molecular Neurosciences at Hannover Medical School for analysis. The staff at Hannover Medical School do not have access to the pseudonym identification list.

d) Your rights under the DSGVO (Datenschutz-Grundverordnung)
You have the right to withdraw your consent to the processing of your biological samples at any time without giving reasons, with effect for the future. The legality of data processing carried out until the withdrawal remains unaffected. Furthermore, all other rights under the GDPR, as described in II.1.f, also apply to the biological samples.

Please note that exercising your rights is only possible until the data are anonymized, as after that, the anonymized data can no longer be attributed to any individual.

**Study title:**	**A Comparison of Two Different Psychotherapy Programs in Persistently Depressed Treatment-Resistant Inpatients**
**Short title:**	**ChangePDD**

Consent Form

I have been thoroughly and clearly informed in a personal conversation by my therapist about the objectives, significance, procedure, risks, and implications of the study. In addition, I have read and understood the patient information text as well as the data protection information for the study. I have had the opportunity to discuss the conduct of the study with my therapist. All my questions have been answered satisfactorily.

Opportunity to document additional questions from the patient or other aspects of the information session:

____

____

____

____

____

I have had sufficient time to make my decision.

I confirm that my consents are given voluntarily. I am also aware that I can withdraw my consents at any time without giving reasons, with effect for the future (either orally or in writing), without any disadvantages for me.

Data Protection Consent

☐ I consent to participate in the above-mentioned clinical study.
(Without this consent, participation in the study is unfortunately not possible.)

☐ I consent to the processing of my personal data, in particular information about my health, within the framework of this clinical trial.
(Without this consent, participation in the study is unfortunately not possible.)

☐ I consent to my treating physician (primary care physician/psychiatrist/neurologist) being informed about my participation in this study.

....
Name and address of physician

....
Name and address of physician

....
Name and address of physician

☐ I consent to the processing of my **biological samples** in accordance with the information provided in Information Sheet Part II.

I have received a copy of the patient information, the consent form, and the insurance documents. One copy will remain at the study site.

....
Patient’s name in block letters

....
Place/Date Signature of patient

I have conducted the information session and obtained the consent of the patient.

....
Name of the physician obtaining consent at the study site in block letters

....
Place/Date Signature of the physician obtaining consent
