# Supplementary material for: Efficacy, moderators and mediators of cognitive behavioural analysis system of psychotherapy (CBASP) versus behavioural activation (BA) in persistently depressed treatment-resistant inpatients: study protocol for the multicentre, randomised controlled changePDD trial
Source: BMJ Open. 2026 Apr 1;16(4):e107051. doi: 10.1136/bmjopen-2025-107051 (PMC13052729; doi:10.1136/bmjopen-2025-107051)
Supplement: online supplemental file 3 [file bmjopen-16-4-s005.pdf]

Patient*inneninformation

**Studientitel:**	**Vergleich zwischen zwei verschiedenen Psychotherapien in der Behandlung von stationären Patient*innen mit lang andauernder Depression**
**Kurztitel:**	**ChangePDD**
**Verantwortliche*r Studienärzt*in:** (Prüfärzt*in)	Name
**Studienzentrum:**	Name der Klinik<br>Adresse<br>Adresse<br>Kontaktdaten (Tel, Fax, E-Mail-Adresse)
**Studienleitung**	Prof. Dr. Eva-Lotta Brakemeier<br>Institut für Psychologie<br>Franz-Mehring-Straße 47<br>17489 Greifswald<br>Telefon: +49-3834-420-3718 / Fax: +49-3834-420-3763<br>E-Mail: eva-lotta.brakemeier@uni-greifswald.de
**Datenschutzbeauftragte*r der Prüfstelle**	
**Datenschutzaufsichtsbehörde des Bundeslandes, in dem Ihre Prüfstelle liegt**	
**Datenschutzbeauftragte*r der Studienleitung**	

Sehr geehrte Patientin, sehr geehrter Patient,

wir möchten Sie hiermit fragen, ob Sie bereit sind, an der nachfolgend beschriebenen klinischen Studie teilzunehmen. Das Kürzel ***ChangePDD*** der Studie symbolisiert unser Ziel: Durch die Studie möchten wir lang andauernde Depressionen (in Englisch: *persistent depressive disorder* = PDD, in Deutschland meist als chronische Depression bezeichnet) durch Psychotherapieprogramme verändern (englisch: *to change*).

Klinische Studien sind notwendig, um Erkenntnisse über die Wirksamkeit von Behandlungsmethoden zu gewinnen oder das Wissen darum zu erweitern.

Die klinische Studie, die wir Ihnen hier vorstellen, wurde von einer Ethikkommission begutachtet. Die Ethikkommission hat keine ethischen Bedenken.

Diese klinische Studie wird in Prüfzentren an mehreren Standorten Deutschlands durchgeführt. Es ist geplant, dass insgesamt 396 Patient*innen daran teilnehmen.

Die Studie wird unter der Leitung von Frau Prof. Dr. Eva-Lotta Brakemeier vom Institut für Psychologie der Universität Greifswald koordiniert und durch die Deutsche Forschungsgemeinschaft (DFG) gefördert.

Ihre Teilnahme an dieser klinischen Studie ist freiwillig. Sie werden in diese Studie also nur dann einbezogen, wenn Sie Ihre Einwilligungen zur Teilnahme sowie zur Verarbeitung Ihrer personenbezogenen Daten schriftlich erteilen. Sie können Ihre Einwilligungen jederzeit ohne Angabe von Gründen mit Wirkung für die Zukunft widerrufen, ohne dass Ihnen daraus irgendwelche Nachteile entstehen.

Sie wurden bereits auf die geplante Studie angesprochen. Die nachfolgenden Informationen sollen Ihnen die Ziele und den Ablauf aufzeigen. Zusätzlich wird ein*e Therapeut*in das Aufklärungsgespräch mit Ihnen führen, indem er*sie Ihnen alle hier genannten Aspekte noch einmal mündlich erläutert. Bitte zögern Sie nicht, alle Punkte anzusprechen, die Ihnen unklar sind. Sie werden danach ausreichend Bedenkzeit erhalten, um über Ihre Teilnahme zu entscheiden.

Kurzdarstellung der Studie

Grund für die Studie: Sie sind an einer lang andauernden bzw. chronischen Depression erkrankt und haben auf andere Therapieversuche bisher nicht ausreichend angesprochen. In dieser Studie geht es darum, die Wirksamkeit eines spezifisch für die chronische Depression entwickelten neueren Psychotherapieverfahrens (genannt „CBASP“) mit einer bereits etablierten Psychotherapie (genannt „BA“) zu vergleichen.

CBASP ist eine relativ neue Psychotherapie, welche für Patient*innen mit einer chronischen Depression und schwierigen Beziehungserfahrungen in der Kindheit entwickelt wurde.

Die bereits bewährte Psychotherapie BA ist eine Variante der etablierten Kognitiven Verhaltenstherapie und hat sich in zahlreichen Studien als wirksam in der Behandlung der Depression erwiesen.

Bisher wurden diese beiden Therapieansätze noch nie miteinander verglichen, so dass wir nicht wissen, welches Therapieprogramm in der Behandlung der chronischen Depression wirksamer ist.

Studienablauf: Wenn Sie sich zur Teilnahme entschließen, dauert die Studie für Sie insgesamt 16 Monate (64 Wochen). Während der ersten 4 Monate (16 Wochen) werden Sie engmaschig in einem teilweise stationären, teilstationären oder ambulanten Setting betreut. 12 Monate danach werden Sie noch einmal zu einer Nachbefragung eingeladen.

Möglicher Nutzen für Sie: Es ist bekannt, dass beide Therapien einen positiven Einfluss auf die chronische Depression haben. Durch Ihre Teilnahme leisten Sie zudem einen wertvollen wissenschaftlichen und gesundheitspolitischen Beitrag, stationäre Psychotherapiekonzepte kurz- und langfristig zu verbessern.

Risiken und Belastungen: Bei jeder psychotherapeutischen Behandlung kann es zu einer vorübergehenden Belastung durch die aktive Auseinandersetzung mit in den Therapiesitzungen besprochenen und bisher möglicherweise vermiedenen Themen kommen. Auch das Ausfüllen der Fragebögen und die Durchführung der Interviews im Verlauf der gesamten Studie (insbesondere zu Beginn) können möglicherweise belastend für Sie sein.
Bitte teilen Sie uns jede Verschlechterung Ihres Gesundheitszustands umgehend mit, unabhängig davon, ob Sie einen Zusammenhang mit der wissenschaftlichen Studie vermuten.

Freiwilligkeit: Es ist Ihre freie Entscheidung, ob Sie an dieser Studie teilnehmen möchten oder nicht. Sie können jederzeit NEIN sagen, sofort oder auch später. Sie brauchen dafür keine Gründe anzugeben und haben keine Nachteile dadurch.
Zusätzlich zur schriftlichen Information werden Sie mündlich aufgeklärt. Fragen Sie den*die Therapeut*in, wenn Sie etwas nicht verstehen. Sie haben anschließend ausreichend Bedenkzeit, um sich für oder gegen eine Teilnahme zu entscheiden. Wenn Sie sich für die Teilnahme entscheiden, unterschreiben Sie bitte die Einwilligungserklärung.

Inhaltsverzeichnis

TEIL I: INFORMATIONEN ZUM ABLAUF DER KLINISCHEN PRÜFUNG UND ZU GESUNDHEITLICHEN ASPEKTEN 5

I. 1. Warum wird diese Studie durchgeführt? 5

I. 1. 1. Erhalte ich die Behandlung auf jeden Fall? 6

I. 1. 2. Wie ist der Studienablauf und was muss ich als Teilnehmer*in wissen? Welche Daten werden während der Studie erfasst? 6

I. 2. Welchen Nutzen hat meine Teilnahme an der Studie? 9

I. 3. Welche Risiken sind mit der Teilnahme an der Studie verbunden? 10

I. 4. Welche anderen Behandlungsmöglichkeiten gibt es? 10

I. 5. Wer darf an dieser klinischen Studie nicht teilnehmen? 11

I. 6. Entstehen für mich durch die Teilnahme an der Studie zusätzliche Kosten? Ist eine Aufwandsentschädigung vorgesehen? 11

I. 7. Bin ich während der Studie versichert? 11

I. 8. Wer sind meine Ansprechpartner während der Studie? 11

TEIL II: INFORMATIONEN ZUM DATENSCHUTZ UND ZU DEN BIOPROBEN 12

II. 1. Was geschieht mit meinen personenbezogenen Daten? 12
- a) Allgemeine Informationen 12
- b) Rechtsgrundlage 12
- c) Verantwortlichkeit 12
- d) Zweck(e) 12
- e) Weitergabe/Empfänger 12
- f) Ihre Rechte gemäß DSGVO (Datenschutz-Grundverordnung) 13

II. 2. Was geschieht mit meinen Bioproben? 15
- a) Verwendung Ihrer Bioproben 15
- b) Lagerung 15
- c) Weitergabe/Empfänger 15
- d) Ihre Rechte gemäß DSGVO (Datenschutz-Grundverordnung) 16

EINWILLIGUNGSERKLÄRUNG 17

Teil I: Informationen zum Ablauf der klinischen Prüfung und zu gesundheitlichen Aspekten

I. 1. Warum wird diese Studie durchgeführt?

Depressive Erkrankungen nehmen bei ca. einem Drittel aller Fälle trotz medikamentöser und/oder psychotherapeutischer Behandlungsversuche einen lang andauernden bzw. chronischen Verlauf. Diese Depressionen werden auch als chronische oder persistierende Depression bezeichnet. Das Kriterium hierfür ist, dass die Depression länger als 2 Jahre anhält.

In der Studie geht es darum, bei Patient*innen mit lang andauernder bzw. chronischer Depression, die auf andere Therapieversuche bisher nicht ausreichend angesprochen haben, die Wirksamkeit eines spezifisch für die chronische Depression entwickelten neueren Psychotherapieverfahrens (genannt „CBASP") mit einer bereits etablierten Psychotherapie (genannt „BA") zu vergleichen. Was verbirgt sich hinter CBASP und BA?

Der etwas sperrige Name **CBASP** steht für „**Cognitive Behavioral System of Psychotherapy**". Der lange Titel weist darauf hin, dass hier Strategien aus verschiedenen Psychotherapie-Richtungen zur Anwendung kommen. CBASP ist eine relativ neue Psychotherapie, welche für Patient*innen mit einer chronischen Depression und schwierigen Beziehungserfahrungen in der Kindheit entwickelt wurde. In ambulanten Studien konnte CBASP bereits seine Wirksamkeit zeigen. In CBASP werden sowohl die Kindheit als auch aktuelle Probleme durch relativ strukturierte Strategien bearbeitet.

BA steht für „**Behavioral Activation**", auf Deutsch „Verhaltensaktivierung". Die bereits bewährte Psychotherapie BA ist eine Variante der etablierten Kognitiven Verhaltenstherapie und hat sich in zahlreichen Studien als wirksam in der Behandlung der Depression erwiesen. In BA wird die Aktivierung als entscheidend für die Behandlung der Depression angesehen. Die Verhaltensaktivierung gelingt insbesondere dann, wenn sich die Aktivitäten aus den Werten (also den Lebensthemen, Lebenszielen) des*der Patient*in ableiten lassen, weshalb die Werte in der Therapie reflektiert werden.

Bisher wurden diese beiden Therapieansätze noch nie miteinander verglichen, so dass wir nicht wissen, welches Therapieprogramm in der Behandlung der chronischen Depression wirksamer ist. Beide Psychotherapieprogramme werden zunächst in einer stationären Behandlung, welche je nach Voraussetzung Ihrer Klinik auch durch eine tagesklinische Phase unterstützt werden kann, und anschließend als ambulante Gruppentherapie durchgeführt, wobei sich die Anzahl der Therapien nicht unterscheidet, sondern lediglich der Inhalt der Therapieprogramme.

Von der Durchführung der vorgesehenen klinischen Studie erhoffen wir uns, die Behandlung von chronisch depressiven Patient*innen künftig noch besser auf die individuellen Bedürfnisse abstimmen zu können und das Wissen über die Wirkweise der beiden Therapieprogramme CBASP und BA zu erweitern.

I. 1. 1. Erhalte ich die Behandlung auf jeden Fall?

Im Falle Ihrer Teilnahme werden Sie auf jeden Fall eines der beiden Therapieprogramme erhalten. Welche Behandlung Sie im Falle Ihrer Teilnahme erhalten, entscheidet der Zufall (dieses Verfahren wird Randomisierung genannt). Die Wahrscheinlichkeit, CBASP oder BA zu erhalten, beträgt jeweils 50 %. Sie erfahren direkt nach der Randomisierung, welche Therapie Sie erhalten.

I. 1. 2. Wie ist der Studienablauf und was muss ich als Teilnehmer*in wissen? Welche Daten werden während der Studie erfasst?

Durch Ihre Teilnahme an der Studie erhalten Sie ein psychotherapeutisches Therapieprogramm. Die Behandlung wird von dem in der Psychotherapie geschulten Behandlungsteam durchgeführt.

Sie werden insgesamt 16 Wochen behandelt, davon sind zunächst 5 Wochen stationäre, im Anschluss dann 5 Wochen teilstationäre/tagesklinische sowie 6 weitere Wochen ambulante Therapie. Zwölf Monate später werden wir Sie zu einer Nachbefragung einladen. Die gesamte Dauer der Studienteilnahme beträgt für Sie 16 Monate (also 64 Wochen).

Während der 5-wöchigen stationären und 5-wöchigen teilstationären Behandlung erhalten Sie <u>pro Woche</u>:

- 2 individuelle Therapiesitzungen (Dauer: 50 Minuten pro Sitzung)
- 2 Gruppentherapie-Sitzungen (Dauer: 100 Minuten pro Sitzung)
- 1 Bezugspflege-Gespräch (Therapeutischer Austausch mit einem*r Krankenschwester*pfleger) (Dauer: 25 Minuten pro Gespräch)
- 1 Bewegungstherapie (Dauer: 75 Minuten pro Therapie)

Während der 6-wöchigen ambulanten Behandlung erhalten Sie <u>pro Woche</u>:

- 1 Gruppentherapie-Sitzung (Dauer: 100 Minuten pro Sitzung)

Dieses Therapieprogramm ähnelt bezüglich der Anzahl und Art der Therapien der Routine-Behandlung, ist jedoch etwas strukturierter und länger:

- Strukturierter, da Sie zunächst stationär, dann teilstationär sowie auch ambulant behandelt werden.
- Länger, da üblicherweise die ambulante Gruppentherapie nicht allen Patient*innen angeboten wird. Die Dauer Ihrer Behandlung wird 4 Monate (also 16 Wochen) betragen.

Zudem können Sie zusätzlich am regulären Therapieprogramm der Station teilnehmen (wie Ergotherapien und Physiotherapien) und zusätzlich auch Medikamente erhalten gemäß eines Behandlungsplans, der auf den aktuellen Leitlinien zur Behandlung der Depression basiert. Um möglichst genaue Rückschlüsse auf den Erfolg Ihrer Therapie im Rahmen der ChangePDD-Studie zu erhalten, ist es leider nicht möglich an allen Therapieprogrammen teilzunehmen. **Bitte sprechen Sie mit Ihrem*Ihrer Therapeut*in, welche der Therapieprogramme Sie vor Ort nutzen können.**

Zusätzlich zu Ihrer therapeutischen Behandlung erfolgen in regelmäßigen Abständen **Studienvisiten**. Die Tabelle 1 (s. unten) veranschaulicht die Art der Visiten sowie den zeitlichen

Ablauf über die gesamte Studie. Wie der Tabelle zu entnehmen ist, gibt es sechs Hauptmesszeitpunkte, welche vor der stationären Aufnahme stattfinden: Voruntersuchung (T0), zu Beginn (T1), nach 5 Wochen (T2) des stationären Aufenthalts, sowie am Ende des stationären oder tagesklinischen Aufenthalts (T3), am Ende der ambulanten Gruppentherapie (T4) sowie der 12-monatigen Verlaufsbeobachtung, also der Nachbefragung (T5).

Zudem werden in der ersten Spalte der Tabelle 1 die verschiedenen Studienvisiten aufgelistet, welche im Folgenden näher beschrieben werden:

– **Basis-Daten und Blutentnahme**: Bei der stationären Aufnahme erheben wir einmalig Ihre allgemeinen demographischen Daten wie zum Beispiel Alter, Geschlecht und Beziehungsstatus (Basis-Daten). Zusätzlich nehmen wir Ihnen ca. 10 ml Blut ab, um einen bestimmten Biomarker im Blut (die sogenannte BDNF-Methylierung) zu bestimmen. Dieser Biomarker misst biologische Prozesse und kann möglicherweise als Indikator (Hinweisgeber) für Umweltbelastungen und Depressionen herangezogen werden. Dieser wird ausgewertet, um herauszufinden, welche Rolle dieser Biomarker bei der Wirksamkeit von psychotherapeutischen Methoden bei der Behandlung der Depression spielt.

– **Lange und kurze klinische Interviews**: Im Rahmen der Interviews werden Sie von einem*r Mitarbeiter*in des Studienteams, welche*r nicht darüber informiert ist, welche der beiden Psychotherapieprogramme Sie erhalten, über Ihr Befinden befragt. Im Rahmen von sogenannten „Kosteninterviews" werden Ihnen zudem Fragen zu Ihrem körperlichen und seelischen Gesundheitszustand und der damit verbundenen Nutzung von verschiedenen Einrichtungen und Angeboten der Gesundheitsversorgung sowie zu Ihrer beruflichen Situation und Wohnsituation gestellt. So können auch die Kosten von Behandlungen und Krankheitstagen etc. mit dem Nutzen der Psychotherapieprogramme ins Verhältnis gesetzt werden. Die langen Interviews werden etwa 90 bis 120 Minuten dauern und die kurzen etwa 15 bis 20 Minuten. Pausen können selbstverständlich jederzeit eingelegt werden.

– **Lange und kurze Fragebogenerhebungen**: Bei diesen Visiten werden Sie gebeten, Fragebögen auszufüllen, die verschiedene Bereiche abdecken (z.B. Depression, weitere Beschwerden, Wohlbefinden, verschiedene Lebensbereiche, Kindheit). Der Umfang der langen Fragebogenerhebung ist größer als in der Routinediagnostik. Maximal werden Sie 23 Fragebögen ausfüllen, was zwischen 60 und 90 Minuten dauern wird. Acht dieser Fragebögen werden während jeder Routinebehandlung erhoben, so dass Sie einen maximalen zeitlichen Mehraufwand von etwa 60 Minuten während einzelner Messzeitpunkte haben werden. Pausen können auch hier natürlich jederzeit eingelegt werden. Bei den kurzen Erhebungen füllen Sie nur wenige Fragebögen aus, was zwischen 10 und 15 Minuten in Anspruch nehmen wird.

- **Fragen zu Nebenwirkungen**: Da Psychotherapie – wie jede Behandlung – auch Nebenwirkungen verursachen kann, ist es uns wichtig, diese zu erfragen. Das Ausfüllen der entsprechenden Fragebögen wird 10 – 15 Minuten dauern.

- **Kurze Beurteilung der Behandlung**: Am Ende einer jeden Behandlungswoche bitten wir Sie, anzugeben, welche Strategien Sie in der letzten Woche angewendet haben und wie sich diese auf Ihre depressive Symptomatik ausgewirkt haben. Die Beantwortung dieser Fragen wird 10 – 15 Minuten dauern.

- **Schrittzähler**: Zudem erhalten Sie für die Dauer des Psychotherapieprogramms (16 Wochen) einen Schrittzähler in Form einer Armbanduhr. Wir bitten Sie, diese während der gesamten 16 Wochen durchgehend zu tragen, so dass automatisch Ihre Schritte gezählt werden (vgl. durchgehender Pfeil in der Tabelle 1). Den Schrittzähler geben wir Ihnen ein weiteres Mal eine Woche vor der letzten Untersuchung aus, um auch hier noch einmal Ihre Schritte zu zählen. Dadurch können wir untersuchen, ob es einen Zusammenhang zwischen Ihrer körperlichen Aktivität und dem Verlauf Ihrer Depression gibt.

- **Außerplanmäßige Visiten**: Nicht immer läuft alles wie geplant. Sollten zwischen Ihrer Voruntersuchung und Ihrer stationären Aufnahme mehr als drei Monate vergehen, so bitten wir Sie, einige Fragebögen und Interviews (siehe „Lange und kurze Fragebogenerhebungen“) in einer zusätzlichen Visite zu wiederholen.

Die Datenerfassung zu Beginn der Studie (T0) ist besonders umfangreich und erfolgt daher an mehreren Tagen. Zur Vergleichbarkeit der Daten erheben wir im selben Umfang auch Daten zu den Zeitpunkten T4 und T5. Hier haben Sie ebenfalls die Möglichkeit, in außerplanmäßigen Visiten die Datenerhebung auf mehrere Tage aufzuteilen.

Tabelle 1: Überblick über die Art und den zeitlichen Ablauf der Studienvisiten

	Vorunter-suchung	Stationäre Behandlung				Tagesklinische Behandlung			Ambulante Behandlung			Verlaufsbeobachtung					
Messzeitpunkt	T0	T1			T2			T3			T4						T5
Woche		1	2	4	5	6	8	10	12	14	16	24	32	40	48	56	64
Basis-Daten	x																
Blutentnahme		x															
Langes klinisches Interview	x	x			x			x			x						x
Kurzes klinisches Interview			x	x		x	x		x	x							
Lange Fragebogen-erhebung	x	x			x			x			x						x
Kurze Fragebogen-erhebung			x	x		x	x		x	x		x	x	x	x	x	
Fragen zu Nebenwirkungen					x			x			x						
Kurze Beurteilung der Behandlung		x	xx	x	x	xx	xx	x	xx	xx	xx						x
Schrittzähler		x	x	x	x	x	x	x	x	x	→ x						x

Die präzise Einhaltung der Untersuchungs- und Therapietermine ist für den Erfolg der Studie von entscheidender Bedeutung. Während der Teilnahme an der ChangePDD-Studie dürfen Sie nicht an einer anderen Studie teilnehmen, es sei denn, diese andere Studie hat einen beobachtenden (nicht-interventionellen) Charakter. Wenn Sie an einer anderen Studie teilnehmen möchten, sprechen Sie vorher bitte unbedingt mit dem*r verantwortlichen Therapeut*in. Während der Dauer der Psychotherapieprogramme (16 Wochen) sollten zusätzliche medikamentöse oder andere psychotherapeutische Behandlungen nur in Absprache mit den Studientherapeut*innen erfolgen.

I. 2. Welchen Nutzen hat meine Teilnahme an der Studie?

Durch Ihre Teilnahme leisten Sie einen wertvollen wissenschaftlichen und gesundheitspolitischen Beitrag, stationäre Psychotherapiekonzepte kurz- und langfristig zu verbessern.

Mögliche Vorteile und Chancen

Folgende Vorteile und Chancen können sich aus der Studienteilnahme für Sie ergeben:

- Keine wochenlange Wartezeit bis zur Aufnahme in die Klinik,
- Wertvolle umfassende Diagnostik Ihrer Probleme während der gesamten Behandlung,
- Eine intensive psychotherapeutische (teil-)stationäre Depressionsbehandlung ,
- Teilnahme an einer ambulanten Gruppentherapie nach Entlassung zur Aufrechterhaltung Ihrer Erfolge und zur Rückfallprävention ,
- Einbindung in eine größere wissenschaftliche Studie mit engmaschiger Betreuung durch geschultes Personal, das stets für Sie ansprechbar ist.

Die Wahrscheinlichkeit ist hoch, dass durch beide intensive Psychotherapieprogramme (CBASP und BA) die chronische Depression positiv beeinflusst und Ihre Probleme gebessert

werden. Es kann jedoch auch vorkommen, dass Sie durch Ihre Teilnahme keinen direkten Nutzen für Ihre psychische Gesundheit haben. Die Gesamtergebnisse dieser wissenschaftlichen Untersuchung sollen dazu beitragen, dass für die Gruppe chronisch depressiver Patient*innen eine wirksame Behandlung gefunden wird und die Zuordnung des*der Patient*in zu dem individuell besser geeigneten Therapieansatz erhöht wird.

I. 3. Welche Risiken sind mit der Teilnahme an der Studie verbunden?

Bei jeder psychotherapeutischen Behandlung kann es zu einer vorübergehenden Belastung durch die aktive Auseinandersetzung mit in den Therapiesitzungen besprochenen und bisher möglicherweise vermiedenen Themen kommen. Das kann unter Umständen zum Auftreten oder einer Zunahme von Suizidalität (Selbsttötungsgedanken oder -plänen) führen. Sie werden regelmäßig zum Thema Suizidalität befragt; zusätzlich bitten wir Sie dringend, sich bei Suizidgedanken jederzeit an das Studienpersonal oder geeignete Notfalldienste zu wenden.

Auch das Ausfüllen der Fragebögen und die Durchführung der Interviews im Rahmen der gesamten Studie (insbesondere zu Beginn) können möglicherweise belastend für Sie sein.

Bitte teilen Sie uns jede Verschlechterung Ihres Gesundheitszustands umgehend mit, unabhängig davon, ob Sie einen Zusammenhang mit der wissenschaftlichen Studie vermuten. Während des stationären Aufenthalts können Sie jederzeit Therapeut*innen Ihres Behandlungsteams aufsuchen und während der ambulanten Phase Studienmitarbeiter*innen (siehe Kontaktdaten am Ende dieser Information) kontaktieren. Wenn die Belastungen zu groß werden, können Sie selbstverständlich ebenfalls zu jeder Zeit das Ausfüllen von Fragebögen, die Durchführung der Interviews oder auch die Teilnahme an der gesamten Studie abbrechen, ohne dass Ihnen daraus irgendwelche Nachteile entstehen. Bei Studienabbruch werden Sie in der Klinik entsprechend der Indikation weiter behandelt wie ein*e „Nicht-Studien-Patient*in“.

Im Rahmen der klinischen Studie wird Ihnen auch einmalig Blut abgenommen (ca. 10 ml). Die Entnahme einer Blutprobe ist in der Regel nur mit einem sehr geringen Risiko verbunden. An der Einstichstelle kann es zu leichten Schmerzen kommen oder es kann ein Bluterguss (blauer Fleck) entstehen, der eventuell einige Tage sichtbar ist. In äußerst seltenen Fällen kann auch die Bildung eines Blutgerinnsels (Thrombose), eine örtlich begrenzte Entzündung oder eine Infektion an der Einstichstelle auftreten, oder es kann zu dauerhaften Schädigungen von Blutgefäßen oder Nerven kommen.

I. 4. Welche anderen Behandlungsmöglichkeiten gibt es?

Ihnen stehen bei Nicht-Studienteilnahme die üblichen Behandlungen in der Klinik zur Verfügung. Bitte fragen Sie Ihre*n Therapeut*in, wenn Sie wissen möchten, welche alternativen Behandlungsmethoden verfügbar sind, sollten Sie nicht an der Studie teilnehmen.

I. 5. Wer darf an dieser klinischen Studie nicht teilnehmen?

An dieser klinischen Studie dürfen Sie nicht teilnehmen, wenn Sie innerhalb des letzten Jahres bereits eine CBASP- oder BA-Therapie erhalten haben oder diese Art von Therapie nicht für Sie geeignet ist.

I. 6. Entstehen für mich durch die Teilnahme an der Studie zusätzliche Kosten? Ist eine Aufwandsentschädigung vorgesehen?

Durch Ihre Teilnahme an dieser Studie entstehen für Sie keine zusätzlichen Kosten. Entsprechend wird Ihnen für die Teilnahme an dieser Studie keine Aufwandsentschädigung gezahlt. Zuzahlungen für gesetzliche versicherte Patient*innen nach §39 (4) SGB V (4) sind von der Studienteilnahme unberührt und können anfallen.

I. 7. Bin ich während der Studie versichert?

Im Mittelpunkt dieser klinischen Studie stehen die zwei Psychotherapieverfahren CBASP und BA. Es werden keine Arzneimittel oder Medizinprodukte verabreicht. Aus diesem Grund ist der Abschluss einer speziellen Probandenversicherung nicht erforderlich.

Allerdings haben wir für Sie eine Wegeunfall-Versicherung abgeschlossen. Diese greift, sollten Sie auf dem Weg zur oder von der Studienteilnahme verunfallen.

Name der Versicherung:

Versicherungsnummer:

Wenn Sie vermuten, dass durch die Teilnahme an der klinischen Studie Ihre Gesundheit geschädigt oder bestehende Leiden verstärkt wurden, müssen Sie dies unverzüglich Ihrem*r Therapeut*in melden.

I. 8. Wer sind meine Ansprechpartner während der Studie?

Sie haben stets die Gelegenheit zu weiteren Beratungsgesprächen mit Ihrem*r Therapeut*in oder dem auf Seite 1 genannten Studienteam, um Fragen im Zusammenhang mit der Studie zu klären. Bitte zögern Sie nicht, Ihre*n Therapeut*in oder das Studienteam zu kontaktieren, falls Sie Fragen haben. Auch Fragen, die Ihre Rechte und Pflichten als Patient*in und Teilnehmer*in der Studie betreffen, werden gern beantwortet.

Teil II: Informationen zum Datenschutz und zu den Bioproben

II. 1. Was geschieht mit meinen personenbezogenen Daten?

a) Allgemeine Informationen
Im Rahmen dieser Studie werden sowohl psychologisch-medizinische Befunde (z.B. aktuelle Untersuchungsbefunde, Medikamente) als auch demographische Daten (Alter, Geschlecht) von Ihnen pseudonymisiert verarbeitet. „**Pseudonymisiert**" bedeutet, dass Ihre personenbezogenen Daten mit einer künstlichen Kennung (bestehend aus Buchstaben und Zahlen) versehen werden, so dass keine unmittelbare Zuordnung zu Ihrer Person möglich ist. Eine Zuordnung der Daten zu Ihrer Person ist nur über eine bei Ihrem*r Therapeut*in hinterlegte Identifikationsliste möglich. Ihre Daten sind gegen unbefugten Zugriff geschützt.
Die Speicherung und Aufbewahrung der personenbezogenen Daten erfolgt in pseudonymisierter Form in Ihrem Studienzentrum sowie bei der verantwortlichen Studienleiterin Prof. Dr. Eva-Lotta Brakemeier (Universität Greifswald).

Nach Beendigung der Studie werden alle pseudonymisierten Daten nach den zu dem Zeitpunkt gültigen Vorschriften gespeichert und aufbewahrt. Diese pseudonymisierten Daten sind nur den Studienmitarbeiter*innen zugänglich. Aktuell beträgt die gesetzlich vorgeschriebene Aufbewahrungsdauer von Studienunterlagen 10 Jahre (§13 Abs. 10 GCP-V).
Des Weiteren werden die personenbezogenen Daten nach Abschluss der Studie in **anonymisierter** Form für weitere wissenschaftliche Forschungen sowie Veröffentlichungen verarbeitet. **„Anonymisiert"** bedeutet, dass die Daten durch Löschung der Pseudonyme nicht mehr Ihrer Person zugeordnet werden können.

b) Rechtsgrundlage
Rechtsgrundlage für Ihre Studienteilnahme sowie die Verarbeitung Ihrer personenbezogenen Daten ist Ihre informierte Einwilligung gemäß EU-Datenschutz-Grundverordnung (Art. 6 Abs. 1 lit. a i.V.m. Art. 9 Abs. 2 lit. a DSGVO). Ohne Ihre ausdrückliche Einwilligung in die Verarbeitung Ihrer personenbezogenen Daten ist eine Teilnahme an dieser Studie leider nicht möglich.

c) Verantwortlichkeit
Verantwortlich im Sinne der Datenschutz-Grundverordnung ist die
Universität Greifswald
vertreten durch

d) Zweck(e)
Der Zweck der Datenverarbeitung dient allein der Planung, Durchführung und Auswertung der Studie ChangePDD. Nähere Informationen zur Studie entnehmen Sie bitte der Patient*inneninformation.

e) Weitergabe/Empfänger
Zugriff auf Ihre Daten haben nur Mitarbeiter*innen der Studie. Diese Personen sind zur Verschwiegenheit und auf den Datenschutz verpflichtet. Die Daten sind vor fremdem Zugriff geschützt. Alle personenbezogenen Daten werden von Ihrem Studienzentrum

pseudonymisiert an Frau Prof. Eva-Lotta Brakemeier (Universität Greifswald, Leiterin der klinischen Studie) übermittelt.

Die von Ihnen im Rahmen der oben genannten klinischen Studie verarbeiteten personenbezogenen Daten (auch die originalen Klardaten) können, soweit erforderlich und gesetzlich erlaubt, durch die zuständige Überwachungsbehörde im Rahmen von Inspektionen oder Beauftragte der Studienleitung (s.g. Auditoren oder Monitore) zur Überprüfung der ordnungsgemäßen Durchführung der klinischen Prüfung in der Prüfstelle eingesehen werden. Diese sind zur Vertraulichkeit verpflichtet, eine Weitergabe der personenbezogenen Daten erfolgt in diesem Zusammenhang nicht.

Wenn Sie die separate Einwilligung zur Information Ihres*Ihrer Hausärzt*in/Psychiater*in/Neurolog*in erteilen, wird diese*r über Ihre Teilnahme an der Studie informiert.

Es findet keine Übermittlung personenbezogener Daten in ein Drittland oder an eine internationale Organisation statt.

f) Ihre Rechte gemäß DSGVO (Datenschutz-Grundverordnung)
Sie haben grundsätzlich folgende Rechte bezüglich Ihrer personenbezogenen Daten, sofern dies nicht aufgrund einer zwischenzeitlich vorgenommenen Löschung der identifizierenden Merkmale zur Entschlüsselung (Pseudonyme) technisch oder aufgrund gesetzlicher Vorschriften unmöglich ist:

Recht auf Widerruf Ihrer Einwilligung
Ebenso wie die Einwilligung zur Teilnahme an der klinischen Studie können Sie auch Ihre Einwilligung in die Verarbeitung Ihrer personenbezogenen Daten jederzeit ohne Angabe von Gründen mit Wirkung für die Zukunft widerrufen. Die Rechtmäßigkeit der bis zum Widerruf erfolgten Datenverarbeitung bleibt unberührt.

Sowohl Ihre Einwilligung in die Studienteilnahme als auch Ihre Einwilligung in die Datenverarbeitung sind Grundvoraussetzungen für Ihre Teilnahme an dieser Studie.

Sie haben weiterhin folgende Rechte
Sie haben das Recht, auf Antrag unentgeltliche Auskunft über Ihre gespeicherten personenbezogenen Daten, deren Herkunft und Empfänger, den Zweck und die Dauer der Datenverarbeitung zu erhalten. Zusätzlich haben Sie unter bestimmten Voraussetzungen das Recht auf Berichtigung, auf Einschränkung der Verarbeitung (z. B. Sperrung), auf Löschung sowie auf die Datenübertragbarkeit Ihrer Daten.

Ferner haben Sie das Recht, bei der zuständigen Aufsichtsbehörde für den Datenschutz eine Beschwerde einzureichen, wenn Sie der Ansicht sind, dass die Verarbeitung Ihrer personenbezogenen Daten nicht rechtmäßig erfolgt. Die zuständige Aufsichtsbehörde finden Sie im Deckblatt der Patient*inneninformation unter dem Punkt *Datenschutzaufsichtsbehörde des Bundeslandes, in dem Ihre Prüfstelle liegt*.

Eine Liste aller in Deutschland und der Europäischen Union zuständigen Datenschutzaufsichtsbehörden finden Sie hier:

https://www.bfdi.bund.de/DE/Infothek/Anschriften_Links/anschriften_links-node.html

Wahrnehmung Ihrer Rechte
Wollen Sie von einem oder mehreren der genannten Rechte Gebrauch machen, kontaktieren Sie bitte Ihre*n Prüfärzt*in.

Bitte beachten Sie, dass die Geltendmachung Ihrer Rechte nur bis zum Zeitpunkt der Anonymisierung möglich ist, da anschließend die anonymisierten Daten keiner Person mehr zugeordnet werden können.

Bei Anliegen zur Verarbeitung personenbezogener Daten und zur Einhaltung der datenschutzrechtlichen Anforderungen können Sie sich auch an die auf dem Deckblatt der Patient*inneninformation benannten Datenschutzbeauftragten der Prüfstelle oder die Studienleitung wenden.

Sie haben grundsätzlich auch das Recht, jederzeit die Studienleitung selbst zu kontaktieren. Bitte wenden Sie sich jedoch im Regelfall an den Prüfärzt*in bzw. den Datenschutzbeauftragten Ihrer Prüfstelle, da aufgrund der Pseudonymisierung Ihre Identität nur an Ihrem behandelnden Zentrum bekannt ist und damit sinnvollerweise weitere Schritte unternommen werden können, bzw. eine unbeabsichtigte Identifikation Ihrer Person durch die Studienleitung vermieden werden kann.

II. 2. Was geschieht mit meinen Bioproben?

Während dieser klinischen Studie werden Ihnen Bioproben (Blutentnahme) entnommen, um einen bestimmten Biomarker im Blut (die sogenannte BDNF-Methylierung) zu bestimmen. Die Proben werden ebenso wie Ihre weiteren personenbezogenen Daten in pseudonymisierter Form aufbewahrt (zur Pseudonymisierung siehe oben II. 1. Buchst. a).

Die Pseudonymisierung Ihrer Bioproben bietet allerdings nicht zwangsläufig den gleichen Schutz wie die Pseudonymisierung der personenbezogenen Daten. Ihre Bioproben beinhalten immer Informationen zu Ihrer Erbsubstanz, die eine Identifizierung ermöglichen könnte. Zudem sind in Ihren Bioproben sensible genetische Informationen gespeichert, die etwa Aussagen zu Verwandtschaftsbeziehungen oder auch Erbkrankheiten zulassen. Ein Identifizierungsrisiko lässt sich nicht völlig ausschließen und steigt, je mehr Daten miteinander verknüpft werden können, z.B. dann, wenn Sie selbst (z.B. zur Ahnenforschung) genetische Daten im Internet veröffentlichen.

Da somit aus Ihren Bioproben personenbezogene Informationen gewonnen werden können, gelten die zuvor unter II. 1. genannten datenschutzrechtlichen Hinweise entsprechend auch für Ihre Bioproben.

a) Verwendung Ihrer Bioproben

Mit Hilfe der Bioproben können biologische Prozesse gemessen und somit möglicherweise als Indikator (Hinweisgeber) für Umweltbelastungen und Depressionen herangezogen werden. Dieser wird ausgewertet, um herauszufinden, welche Rolle dieser Biomarker bei der Wirksamkeit von psychotherapeutischen Methoden bei der Behandlung der Depression spielt.

Erste Studienergebnisse deuten bereits darauf hin, dass dieser Biomarker für das Ansprechen auf eine Behandlung relevant sein könnte. Daher planen wir, auf diesen Ergebnissen aufbauend, sogenannte genomweite Assoziationsstudien sowie Kandidatengenstudien (Studien, die dazu dienen, diesen potentiellen Biomarker bestimmten Verläufen der Depression zuzuordnen). Hiermit könnte es künftig möglich sein, computerbasierte Algorithmen zu nutzen, um anhand dieses Biomarkers Patient*innen jene Therapie zuzuordnen, die am besten zu den individuellen Bedürfnissen passt.

b) Lagerung

Die Lagerung erfolgt in der Biobank der Medizinischen Hochschule Hannover. Die Bioproben werden entsprechend der Richtlinie der Medizinischen Hochschule Hannover 5 Jahre lang in pseudonymisierter Form vorgehalten und anschließend anonymisiert (zur Anonymisierung siehe oben II. 1. Buchst. a).

c) Weitergabe/Empfänger

Die Blutproben werden ausschließlich in pseudonymisierter Form für diese klinische Studie verarbeitet und zur Untersuchung ins Labor für molekulare Neurowissenschaften der Medizinischen Hochschule Hannover geschickt. Die Mitarbeitenden der Medizinischen Hochschule Hannover haben keinen Zugang zur Identifikationsliste der Pseudonyme.

d) Ihre Rechte gemäß DSGVO (Datenschutz-Grundverordnung)

Selbstverständlich haben Sie das Recht, Ihre Einwilligung in die Verarbeitung Ihrer Bioproben jederzeit ohne Angabe von Gründen mit Wirkung für die Zukunft widerrufen. Die Rechtmäßigkeit der bis zum Widerruf erfolgten Datenverarbeitung bleibt unberührt. Ferner gelten auch für die Bioproben alle weiteren Rechte gemäß DSGVO, wie unter II. 1. f) genannt.

Bitte beachten Sie, dass die Geltendmachung Ihrer Rechte nur bis zum Zeitpunkt der Anonymisierung möglich ist, da anschließend die anonymisierten Daten keiner Person mehr zugeordnet werden können.

**Studientitel:**	**Vergleich zwischen zwei verschiedenen Psychotherapien in der Behandlung von stationären Patient*innen mit lang andauernder Depression**
**Kurztitel:**	**ChangePDD**

Einwilligungserklärung

Ich bin in einem persönlichen Gespräch durch den*die Therapeut*in ausführlich und verständlich über Ziele, Bedeutung, Ablauf, Risiken und Tragweite der Studie aufgeklärt worden. Ich habe darüber hinaus den Text der Patient*inneninformation sowie die Datenschutzhinweise zur Studie gelesen und verstanden. Ich hatte die Gelegenheit, mit dem*der Therapeut*in über die Durchführung der Studie zu sprechen. Alle meine Fragen wurden zufriedenstellend beantwortet.

Möglichkeit zur Dokumentation zusätzlicher Fragen seitens des*der Patient*in oder sonstiger Aspekte des Aufklärungsgesprächs:

____

____

____

____

____

Ich hatte ausreichend Zeit, mich zu entscheiden.

Ich bestätige, dass meine Einwilligungen auf Freiwilligkeit beruhen. Zudem ist mir bekannt, dass ich jederzeit meine Einwilligungen ohne Angabe von Gründen mit Wirkung für die Zukunft widerrufen kann (mündlich oder schriftlich), ohne dass für mich daraus Nachteile entstehen.

Datenschutzrechtliche Einwilligung

☐ Ich willige ein, an der oben genannten klinischen Studie teilzunehmen.
(Ohne diese Einwilligung ist eine Teilnahme an der Studie leider nicht möglich.)

☐ Ich willige ein, dass im Rahmen dieser klinischen Prüfung personenbezogene Daten von mir, insbesondere Angaben über meine Gesundheit, verarbeitet werden.
(Ohne diese Einwilligung ist eine Teilnahme an der Studie leider nicht möglich.)

☐ Ich willige ein, dass mein*e behandelnder Ärzt*in (Hausärzt*in/Psychiater*in/Neurolog*in) über meine Teilnahme an dieser Studie informiert wird.

....
Name und Anschrift des*r Ärzt*in

....
Name und Anschrift des*r Ärzt*in

....
Name und Anschrift des*r Ärzt*in

☐ Ich willige ein, dass meine **Bioproben** gemäß den Angaben der Informationsschrift Teil II 2. verarbeitet werden.

Ein Exemplar der Patient*innen-Information und der Einwilligungserklärung sowie die Versicherungsunterlagen habe ich erhalten. Ein Exemplar verbleibt in der Prüfstelle.

....
Name des*r Patient*in in Druckbuchstaben

....
Ort/Datum | Unterschrift des*r **Patient*in**

Ich habe das Aufklärungsgespräch geführt und die Einwilligung des*r Patient*in eingeholt.

....
Name des*r aufklärenden Ärzt*in der Prüfstelle in Druckbuchstaben

....
Ort/Datum | Unterschrift des*r aufklärenden **Ärzt*in der Prüfstelle**
