# Supplementary material for: Efficacy, moderators and mediators of cognitive behavioural analysis system of psychotherapy (CBASP) versus behavioural activation (BA) in persistently depressed treatment-resistant inpatients: study protocol for the multicentre, randomised controlled changePDD trial
Source: BMJ Open. 2026 Apr 1;16(4):e107051. doi: 10.1136/bmjopen-2025-107051 (PMC13052729; doi:10.1136/bmjopen-2025-107051)
Supplement: Supplementary file 7 [file bmjopen-2025-107051.reviewer_comments.pdf]

PEER REVIEW HISTORY

BMJ Open publishes all reviews undertaken for accepted manuscripts. Reviewers are asked to complete a checklist review form and are provided with free text boxes to elaborate on their assessment. These free text comments are reproduced below.

ARTICLE DETAILS

Title (Provisional)

Efficacy, moderators and mediators of Cognitive Behavioral Analysis System of Psychotherapy (CBASP) versus Behavioral Activation (BA) in persistently depressed treatment-resistant inpatients: Study protocol for the multi-center, randomized controlled ChangePDD trial

Authors

Brakemeier, Eva-Lotta; Klein, Jan Philipp; Zimmermann, Johannes; Hollandt, Maike; Reinhard, Matthias Alexander A.; Boger, Sabrina; Daldrup, Luisa; Eldem, Luk; Gebhardt, Philippa; Heinrich, Susanne; Hirsmueller, Matthias; Millerowski, Jacob; Richter, Martina; Ridderbusch, Isabelle; Suerig, Svenja; Schroeter, Lennart; Velten-Schurian, Kerstin; Engeli, Stefan; Witte, Amelie; Bajbouj, Malek; Fallgatter, Andreas J; Kahl, KG; Kircher, Tilo; Köhler, Stephan; Philipsen, Alexandra; Walter, Martin; Wolf, Johannes; Guhn, Anne; Schweiger, Ulrich; Hoyer, Jürgen; Gutzmer, Dennis; Mauersberger, Stephan; Demir, Selin; Stapel, Sarah; Hallgans, Jonas; Schüle, Cornelius; Frischholz, Christian; Heitland, Ivo; Rek, Stephanie; Jobst, Andrea; Kluge, Ina; Lux, Silke; Opel, Nils; Orlowksi, Svenja; Reinert, Christina; Voelz, Henrike; Berwian, Isabel M.; Frieling, Helge; Maier, Hannah B.; Walter, Henrik; Fassbinder, Eva; Kaiser, Tim; Kanter, Jonathan; Swan, John; Cuijpers, Pim; Hautzinger, Martin; Sterzer, Philipp; Padberg, Frank

---

This article was not externally reviewed at BMJ Open. Protocols that have been independently assessed prior to submission to BMJ Open are usually fast-tracked to publication on the grounds that further substantial changes will not be possible. This independent assessment will usually be external, independent review for both a substantial grant award from a non-commercial or government funder and ethics approval.
